# Fluorescent Antibiotics: Bridging Diagnostic and Therapy in the Fight against Bacterial Infections

**DOI:** 10.1002/smsc.202500138

**Published:** 2025-05-20

**Authors:** Tan Phuoc Ton, Richard Bright, Vi Khanh Truong, Krasimir Vasilev

**Affiliations:** ^1^ Biomedical Nanoengineering Laboratory College of Medicine and Public Health Flinders University Bedford Park SA 5042 Australia; ^2^ Department of Biomedical Engineering and Biotechnology Khalifa University P.O. Box: 127788 Abu Dhabi UAE

**Keywords:** bacterial infections, diagnostics, fluorescent antibiotics, fluorescent probes, imagings

## Abstract

Infections caused by pathogenic bacteria pose a significant threat to human health. Fluorescent antibiotics present an innovative material class that combines diagnostic capabilities with therapeutic effects. Fluorescent antibiotics offer a transformative strategy by combining precise visualization of bacterial activity with targeted antimicrobial action, addressing key challenges like drug resistance and rapid pathogen detection in diagnostics and treatment. This review highlights recent advances in the development of fluorescent antibiotics, focusing on chemical strategies such as click chemistry, amide bond formation, solid‐phase peptide synthesis, and epoxy‐amine addition. Also, the practical applications of these fluorescent probes, ranging from imaging, rapid detection, and real‐time visual tracking of pathogenic bacteria to the detailed study of molecular dynamics at the cellular level are discussed. The opportunity to combine detection and treatment of infectious diseases underscores the significant promise of these probes, particularly in the face of escalating antimicrobial resistance. However, despite the promise, most studies are confined to laboratory settings. To move forward, continued innovation and rigorous clinical validation will be essential to translating these promising molecular tools from laboratory research to impactful clinical applications.

## Graphical abstract

This review critically evaluates fluorescent antibiotics as promising agents combining diagnostic precision with therapeutic efficacy. It highlights advances in probe design, chemical conjugation strategies, real‐time imaging applications, selective pathogen detection, and targeted therapy. The potential to combat antimicrobial resistance is discussed, alongside the need for clinical validation and translational studies to realize their full potential.

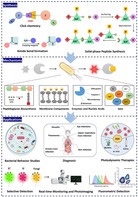

## 1 Introduction

The bacterial infection crisis, currently the second leading cause of death worldwide, demands immediate attention and action.^[^ ^1^ ^]^ Antibiotics, one of the most significant medical breakthroughs of the 20th century, have been pivotal in combating bacterial infections and reducing mortality worldwide.^[^ ^2^ ^]^ Furthermore, antibiotics revolutionized many other areas of healthcare by enabling modern procedures such as cancer treatments, organ transplants, and open‐heart surgeries. Nonetheless, according to the Center for Disease Control (CDC), approximately half of antibiotic prescriptions in hospitals and nursing home settings were deemed inappropriate or unnecessary, primarily attributed to delays and inaccuracies in diagnosis.^[^ ^3^ ^]^ Overtime, the inappropriate and excessive use of antibiotics has contributed to the emergence of multidrug‐resistant (MDR) bacteria.^[^ ^4^ ^]^ While some bacteria are naturally resistant to certain antibiotics, MDR bacteria usually develop resistance through adaptation when exposed to these drugs. Resilient bacteria resist antibiotics by modifying drug targets, expelling antibiotics via efflux pumps, degrading antibiotics with enzymes, reducing cell permeability by downregulating porins, and acquiring resistance genes through horizontal gene transfer.^[^ ^5^ ^]^

The CDC estimated that MDR infections result in $20 billion in direct healthcare costs and cause at least 23 000 deaths annually.^[^ ^6^ ^]^ It is predicted that by 2050, MDR could lead to 10 million deaths per year globally and incur economic losses of up to $100 trillion if no action is taken.^[^ ^1^, ^7^ ^]^ In addition, the pharmaceutical industry has increasingly withdrawn its investment in developing new antibiotics due to increasing costs, which threatens to escalate the issue further^[^ ^2^, ^8^ ^]^ and return humanity to a pre‐antibiotic era.^[^ ^9^ ^]^

Given the possibility that new antibiotic development may not keep pace with the rapid evolution of bacteria, the need for better infection control via advancing detection methods becomes imperative. Early identification of the primary causes of infections is a practical approach to prevent severe outcomes. Conventional bacterial detection methods involve solid or liquid culture followed by biochemical analysis. While simple and cost‐effective, these methods are highly time‐inefficient, often taking days to weeks for a culture to grow.^[^ ^10^ ^]^ Current methods for rapid bacterial identification include enzyme‐linked immunosorbent assay (ELISA), polymerase chain reaction (PCR), and lateral flow assays. The primary drawback of these methods lies in their considerable expense and the necessity for skilled personnel, restricting their accessibility, particularly in low‐income regions.^[^ ^11^, ^12^ ^]^ One of the emerging bacterial detection methods is biosensors, analytical devices that convert biological responses and amplify them into electrical signals, offering a cutting‐edge alternative to molecular bacterial detection techniques, delivering specific, sensitive, and real‐time monitoring with straightforward sample preparation and rapid detection time. However, their performance is frequently constrained by factors such as high costs and sensitivity to variations in temperature, pH, and other operational conditions.^[^ ^12^, ^13^ ^]^

Fluorescent‐based probes have emerged as cost‐effective and powerful tools for detecting bacterial infections, including MDR bacteria.^[^ ^14^, ^15^ ^]^ These probes can detect bacteria even at lower concentrations by offering real‐time identification with high sensitivity and selectivity. Their efficiency in providing quick and accurate results is a testament to their effectiveness.^[^ ^15^, ^16^ ^]^ The streamlined process, requiring minimal sample preparation and quick incubation, enables faster and more efficient diagnostics than traditional methods. Fluorescent probes are highly adaptable and comprise three key components: a chromogenic element for fluorescence, a connecting element for stability, and an identifying element for specificity.^[^ ^15^ ^]^ Innovations such as antibiotic‐conjugated dyes and nanoparticles (NPs) have expanded their utility in diverse biomedical applications. With their speed, precision, and versatility, fluorescent‐based probes are poised to revolutionize infection diagnostics and combat antimicrobial resistance, addressing a critical challenge in modern healthcare.

Via review, we aim to provide a comprehensive overview of the current advancements in fluorescently tagged antibiotics, addressing the rapidly increasing interest in developing and applying such probes in recent years. In addition to filling the need for an up‐to‐date field analysis, this review provides and elucidates different strategies for designing fluorescent antibiotics, including the selection of fluorophores, targeted elements, fluorescence mechanisms, and modes of action and proof‐of‐concept chemical synthesis. Furthermore, this review explores the in vitro, in vivo, ex vivo, and emerging clinical applications of fluorescently tagged antibiotics in healthcare diagnostics, treatment, and biomedical research. It also highlights the gaps and limitations in current technologies, offering insights to guide future improvements and innovations. By providing a robust reference point, this review aims to empower the continued advancement of fluorescent‐tagged antibiotics in combating bacterial infections and addressing critical challenges in modern medicine.

## 2 Strategies for Designing Fluorescent‐Tagged Antibiotics

Fluorescent antibiotics are designed to combine the ability to target bacteria with the functionality of diagnostic visualization. The design process focuses on maintaining the biological activity of the antibiotic while optimizing the fluorescence properties of the conjugate; thereby, careful consideration regarding the choice of antibiotic, fluorophore, and suitable synthetic strategies is required. For instance, the functional groups in the chemical structure of the antibiotic are carefully selected for modification to ensure that they do not interfere with their ability to bind to bacterial targets. Simultaneously, a fluorophore is selected for its high fluorescence intensity, antibiotic compatibility, and stability in biological environments. Factors such as size, charge, and light excitation/emission properties are essential to balance effective targeting and reliable imaging. Furthermore, many physicochemical properties of the precursor elements, including molecular weight, polarity, and sensitivity to light, pH, and temperature, strongly affect the synthetic strategies, which aim to produce the highest product purity with minimal reagents and solvents.

### 2.1 Selection of Antibiotics

**Figure 1:**
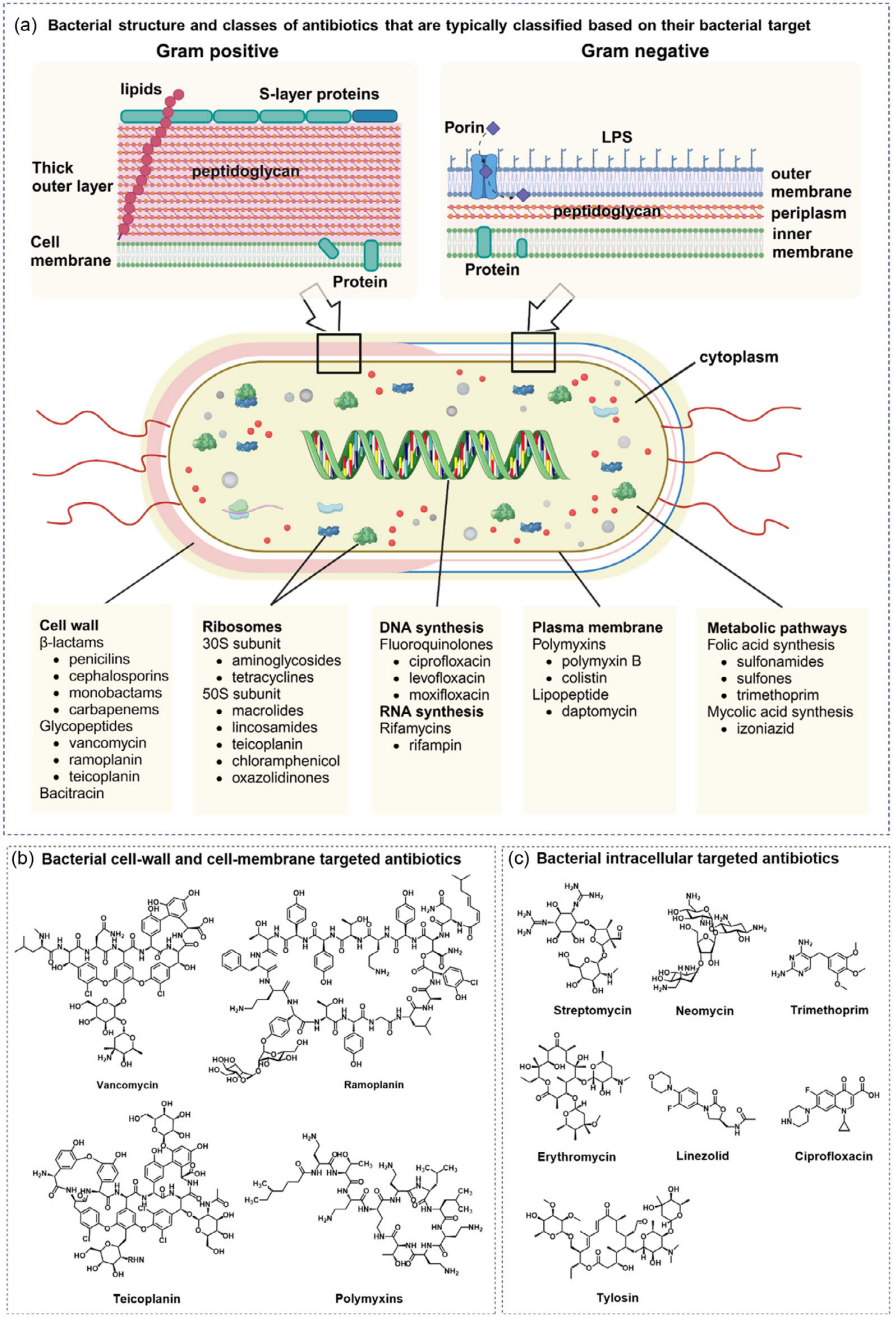
Bacterial structures, classes, and chemical structure of antibiotics targeting bacteria. a) Bacterial architecture highlighting key targets: Gram‐positive bacteria with a thick peptidoglycan and Gram‐negative with a thinner peptidoglycan layer and an outer membrane containing LPS and porins. Intracellular targets include proteins, DNA, and RNA. b) Chemical structures of antibiotics targeting cell walls and membranes. c) Chemical structures of antibiotics targeting intracellular bacterial components.

The precise, selective action of antibiotics against bacteria is what makes them invaluable in infection treatment, as they effectively target bacterial cells while being relatively safe for human host cells.^[^ ^17^ ^]^ The choice of antibiotics depends strongly on bacterial structures; in many cases, the differences between Gram‐positive and Gram‐negative bacteria are the main consideration (**Figure** **1**a). The key distinction lies in the bacterial cell wall: Gram‐positive bacteria feature a thick peptidoglycan (PG) layer, while Gram‐negative bacteria have a thinner PG layer and an additional outer membrane.^[^ ^18^, ^19^, ^20^, ^21^, ^22^ ^]^ The thick PG layer of Gram‐positive bacteria more readily absorbs antibiotics, making them more susceptible to drugs targeting PG synthesis. In contrast, the additional membrane in Gram‐negative bacteria serves as a barrier, making them more resistant to antibiotics that are effective against Gram‐positive bacteria. However, this membrane is covered by lipopolysaccharides (LPS) and porins, which can be targeted by antibiotics or render drug entry into the cell. Some antibiotics are engineered to target these distinct features of bacterial cell walls and membranes, allowing them to discriminate Gram‐positive against Gram‐negative bacteria precisely. In contrast, some antibiotics are drawn to internal bacterial structures like proteins, ribosomes, and nucleic acids. Others are designed to target metabolic pathways, such as folic acid and mycolic acid synthesis.^[^ ^21^, ^23^, ^24^ ^]^

Vancomycin (Van), a member of the glycopeptide class, is the oldest and most used antibiotic that inhibits a late stage in bacterial cell wall PG synthesis (**Table** **1** and 3). By binding to the terminal D‐alanyl‐D‐alanine residues of PG precursors, Van disrupts PG cross‐linking, compromising cell wall structure integrity. This mechanism makes glycopeptides highly effective against gram‐positive bacteria, including methicillin‐resistant *Staphylococcus aureus* (MRSA). Similar glycopeptides, such as teicoplanin and ramoplanin, are also built to suppress bacterial cell wall formation through analogous interactions.^[^ ^25^, ^26^, ^27^ ^]^

**Table 1:** Fluorescent antibiotics targeting the cell wall of Gram‐positive bacteria.

Compound name	Synthesis	Ex/Em wavelengths	Targeted species	Applications	References
Fluorescent dyes					
Van–FL Van–BDP Ramo‐4Fl Ramo‐10Fl Ramo‐4BDP	NHS ester‐amine		*B. subtilis*	Imaging peptidoglycan biosynthesis	[139]
Van–porphyrin	HBTU	Ex = 535/50 nm Em = 610/75 nm	VSE, VRE, and MRSA	Fluorescent imaging and photodynamic inactivation of vancomycin‐sensitive and vancomycin‐resistant bacterial strains	[122]
Vanco‐800CW	NHS ester‐amine	745/>785 nm (840 nm)	*S. aureus* and *S. epidermidis*	In vivo imaging of bacterial infections in mice; discrimination between bacterial infection and sterile inflammation; detection of bacterial biofilms on implanted medical devices in human cadavers	[115]
PTPVan		458/558 nm	*S. aureus, S. epidermidis*, and MRSA	Detection of Gram‐positive bacteria through color change and fluorescence response Staining Gram‐positive bacteria	[116]
Rho‐FF‐Van Rho‐GG‐Van	Solid‐phase peptide and liquid‐phase synthesis	540/≈580 nm	MRSA	In vivo imaging of Gram‐positive bacterial infections	[159]
Van–BODIPY Van–IRdye800CW Van–IRdye700DX	HATU or HBTU		*S. aureus* and *E. faecium*	In vitro detection of Gram‐positive bacterial biofilms	[123]
Merocy–Van	Reductive amination	590/620 nm	*S. aureus, S. epidermidis, S. pneumoniae*, and MRSA	Rapid, specific in situ detection of Gram‐positive bacteria in ex vivo human lung models	[135]
Van‐4‐NBD	Reductive amination	480/≈540 nm	*S. aureus* and *S. epidermidis*	Rapid detection and imaging of Gram‐positive bacterial pathogens in ocular species	[137]
Vanco‐800CW Vancomycin‐BODIPY FL			*S. aureus*	Bacteria‐targeted fluorescence imaging of fracture‐related infections	[166]
Van‐8C‐Tz‐NBD Van‐8C‐Tz‐DMACA	CuAAC	475/535 nm 375/480 nm	*S. aureus* and *E. coli*	Detection and visualization of Gram‐positive bacteria Measuring outer membrane permeabilization of Gram‐negative bacteria	[56]
Aggregation‐induced emission					
AIE‐2Van	CuAAC	405/650	VRE *and B. subtilis*	Selective recognition, direct naked‐eye detection, and image‐guided photodynamic killing of Gram‐positive bacteria	[91]
Nanoparticles					
SiNPs–Van	EDC/NHS	405/480 nm 460/520 nm	*S. aureus*	Prolonged in vivo fluorescence tracking and therapy of *S. aureus* infections	[108]
DCNPs–Van/Cu_2−*x*_Se	EDC/NHS Electrostatic binding of Cu_2−x_ Se and vancomycin‐modified NaGdF4:Nd,Yb@NaGdF4 DCNPs	808/1060 nm	*S. aureus*	Enzyme‐independent bacteria‐activated NIR‐II fluorescence for in vivo imaging of bacterial infections	[161]
Van–CDs	EDC/NHS	320/435 nm	*S. aureus, B. subtilis*, and *Listeria monocytogenes* (*L. monocytogenes*)	Rapid and sensitive detection of Gram‐positive bacteria in complex samples	[109]
Van‐modified OSiNDs	Epoxy‐amine	511/528 nm	*S. aureus*, MRSA*, and E. coli*	High‐quality imaging and elimination of multidrug‐resistant bacteria and their biofilms	[138]
Van QD‐CPP QD‐CPP‐Van	Electrostatic interaction		*S. aureus, P. aeruginosa*, and *E. coli*	Eradicating biofilm formation and addressing bacterial resistance	[145]
Vanc‐TCO or dapt‐TCO and MFNPs	Bioorthogonal chemistry		*S. aureus, S. pneumoniae, S. epidermidis, and E. faecalis*	Selective and intracellular detection of Gram‐positive bacteria	[99]

Fluorescent polymyxins are the most extensively studied fluorescent antibiotics for targeting bacterial cell membrane (**Table** **2** and **3**). Polymyxins are cyclic polypeptides that interact with the LPS and phospholipids of the outer cell membrane of Gram‐negative bacteria. This damages the membrane integrity, resulting in the loss of intracellular components and bacterial cell death. They are particularly effective against MDR Gram‐negative bacteria, such as carbapenem‐resistant strains, but their toxicity limits their use to last‐line therapy.^[^ ^28^, ^29^, ^30^, ^31^ ^]^

**Table 2:** Fluorescent antibiotics targeting cell membrane of Gram‐negative bacteria.

Compound name	Synthesis	Ex/Em wavelengths	Targeted species	Applications	References
Fluorescent dyes					
Dansyl[Lys]^1^polymyxin B_3_	Solid‐phase peptide synthesis Hinsberg reaction	340/485 nm	*E. coli, A. baumannii, P. aeruginosa, Salmonella enterica* (*S. enterica*), and *K. pneumoniae*	Quantitatively measuring the binding interactions between polymyxin and LPS or whole bacterial cells	[126]
Dansyl–polymyxin B	Solid‐phase peptide synthesis	360–400/457–507 nm	*P. aeruginosa, A. baumannii*, and *K. pneumoniae*, including polymyxin‐resistant strains	Imaging polymyxins penetration and localization within Gram‐negative bacterial cells	[127]
PmB MIPS‐9541 MIPS‐9542 MIPS‐9543 MIPS‐9544	Solid‐phase peptide synthesis	405/470‐560 nm	Rat kidney tubular cells	Examining the localization of polymyxins in rat renal tubular cells to gain further insights into the mechanism of polymyxin‐induced nephrotoxicity	[128]
NBD–PMX		450/≈550 nm	*P. aeruginosa, K. pneumoniae, E. coli, Haemophilus influenzae* (*H. influenzae*)*, A. baumannii*, and *Stenotrophomonas maltophilia* (*S. maltophilia*)	Rapid in situ identification of Gram‐negative bacteria and cellular infiltrates in distal human lungs in real time	[164]
meroSO‐PMX meroBA‐PMX	HSPyU	590/630 nm 570/600 nm	*P. aeruginosa* and *E. coli*	Selective detection of Gram‐negative bacteria in wash‐free protocol	[136]
TLipPmB‐23 TlipP2K‐23	Synthesis	630/650 nm	*E. coli, P. aeruginosa*, and *Proteus mirabilis* (*P. mirabilis*)	Rapid and selective detection of Gram‐negative bacteria via fluorescence activation	[163]
PMS‐DNS PMS‐Cy‐NO_2_	Solid‐phase peptide synthesis	405/450 nm 745/800 nm	*K. pneumonia, A. baumannii, P. aeruginosa, E. cloacae*, and *E. coli*, but no PMX resistance strains	Selective labeling of Gram‐negative bacteria in complex biological samples and in vivo models	[129]
COL–Cy3	NHS ester‐amine	540/570 nm	*E. coli, P. aeruginosa*, and *K. pneumoniae*	Rapid (≈10 min), direct, and universal detection of Gram‐negative bacteria in complex environments, such as samples including multiple targets and samples including other biomolecules or cells from body fluids	[162]
BODIPY‐labeled colistin		585/535 nm	*P. aeruginosa* biofilm	Assessing antibiotic entry into biofilm	[167]
BODIPY–daptomycin Rhodamine–daptomycin			*S. epidermidis* biofilm	Determining the time course of diffusive penetration of daptomycin into large, dense cluster of *staphylococcal* biofilm	[168]
Aggregation‐induced emission					
AIE‐DCM‐2polymyxinB	Solid‐phase peptide synthesis Thiol‐maleimide coupling reaction	439/>650 nm	*E. coli* and *Salmonella* *Enteritidis* (*S. enteritidis*)	Selective visualization and effective killing through photodynamic treatment of Gram‐negative bacteria	[93]
Nanoparticles					
PMB‐UCNPs	EDC/NHS	980/801 nm	*E. coli*	Selective detection and fluorescence imaging of Gram‐negative bacteria	[111]
PMB–CON	Click chemistry reaction	520/634	*kanaR E. coli*	Selective identification, targeted imaging, and synergetic photodynamic killing of *kanaR E. coli* in one treatment	[95]
Colis–QDs	EDC/NHS	400/575	*E. coli*	Fast (less than 15 min), sensitive method for *E. coli* detection in complex samples without the need for pre‐enrichment	[110]
Nitrogen‐doped CDs@colistin	Solid‐phase synthesis	360/447 nm	*E. coli*	Ultrasensitive detection of *E. coli* in human urine, apple juice, and tap water Selective imaging of *E. coli*	[169]

**Table 3:** Fluorescent antibiotics targeting cell wall and membrane.

Compound name	Synthesis	Ex/Em wavelengths	Targeted species	Applications	References
Fluorescent dyes					
8AzKdo Vanco‐Cy3	NHS ester‐amine	547/572 nm 550/570 nm	*E. coli*	Two‐color fluorescence imaging of Gram‐negative and Gram‐positive bacteria in the mouse intestines	[117]
Vanco‐Cy5 PxB‐Cy3	NHS ester‐amine	650/670 nm 550/570 nm	*E. coli, P. aeruginosa*, and *S. enterica*; *S.* aureus, *Streptococcus gordonii* (*S. gordonii*), and *E. faecalis*	Two‐color imaging of Gram‐positive and Gram‐negative bacteria in various living microbiotas, including mouse gut, human oral, soil, and crude oil microbiotas Staining bacteria on heat‐fixed sputum smear	[118]
Nanoparticles					
CaS–TEC NPs SrS–PMB NPs	EDC/NHS	645/980 nm 465/980 nm	*E. coli* and *P.* aeruginosa; S*. aureus* and B. subtilis	Selective imaging of Gram‐positive and Gram‐negative bacteria in co‐infected mice models Deep tissue imaging	[112]
csYb, Er, Ce–Van DCNPs csNd, Yb, PMB DCNPs	EDC/NHS	980/1525 nm 808/980 and 1060 nm	*E. coli* and *P.* aeruginosa; S*. aureus, B. subtilis*, and *Enterococcus faecalis* (*E. faecalis*)	Selective binding and multiplexed imaging of Gram‐positive and Gram‐negative bacterial infection in vivo	[113]
Van–Pdots PB–Pdots	EDC	475/535 nm	*P. aeruginosa*; *S. aureus*	Detection, imaging, and photo‐killing of bacteria	[114]
BA–CDs PM–CDs Van–CDs	One‐pot method	350/455 nm	*E. coli, Desulfovibrio desulfuricans* (*D. desulfuricans*)*, Staphylococcus sciuri* (*S. sciuri*)*, L. monocytogenes, S. aureus*, and *P. aeruginosa*	Fluorescent sensor array for rapid and effective discrimination of bacterium types	[141]

The primary mechanism of action of antibiotics that selectively target intracellular structures of bacteria is to impede protein synthesis through binding to different subunits of the bacterial ribosome (**Table** **4**). Aminoglycosides, such as streptomycin, neomycin, and tylosin, attach to the 30S subunit and cause misreading of bacterial RNA.^[^ ^32^ ^]^ Streptomycin is effective against tuberculosis and other infections, whereas neomycin is applied topically to prevent wound and burn infections.^[^ ^33^ ^]^ Erythromycin and linezolid both bind to the 50S subunit but at different sites. Erythromycin is a macrolide that blocks the translocation of the peptide chain. It is utilized to treat respiratory, skin, and chlamydial infections. Linezolid, an oxazolidinone that interferes with the initiation complex formation, treats serious infections caused by MRSA and other resistant bacteria.^[^ ^34^, ^35^ ^]^ Fluoroquinolones are antibiotics that suppress bacterial DNA synthesis by targeting DNA gyrase and topoisomerase IV. These enzymes unwind and separate the DNA strands during replication and transcription. Fluoroquinolones are broad‐spectrum antibiotics that can treat urinary, respiratory, and skin infections.^[^ ^36^ ^]^ Trimethoprim, similar in activity to fluoroquinolones, targets bacterial DNA synthesis by inhibiting dihydrofolate reductase (DHFR), an essential enzyme involved in the synthesis of nucleotides. Trimethoprim is commonly used with sulfamethoxazole (as co‐trimoxazole) to enhance its antibacterial spectrum, effectively treating urinary tract infections, diarrhea, and certain types of pneumonia.^[^ ^37^ ^]^

**Table 4:** Fluorescent antibiotics targeting intracellular bacteria.

Compound name	Synthesis	Ex/Em wavelengths	Targeted species	Applications	References
BODIPY‐erythromycin (macrolides)	NHS ester‐amine	485/530 nm	*E. coli*	Measures the binding of fluorescently labeled erythromycin to 70S ribosomes from *Escherichia coli* and the displacement of erythromycin from these ribosomes	[119]
BODIPY‐erythromycin	NHS ester‐amine	504/513 nm	*E. coli*	Ultrahigh‐throughput screening to identify novel ribosome inhibitors	[120]
Fluorescein‐neomycin (F‐neo)	NHS ester‐amine	490/517 nm	*E. coli*	Measure the binding affinity of various drugs to the ribosomal A site	[121]
Lz‐tz‐DMACA Lz‐tz‐NBD	CuAAC	400/490 nm 475/550 nm	*S. pneumoniae, E. faecalis, E. faecium*, MRSA, and VRE	Explore antimicrobial structure–activity relationships; imaging of Gram‐positive bacteria	[87]
4C‐Tz‐NBD 4C‐Tz‐DMACA 4C‐Tz‐DNS	CuAAC	475/545 nm 380/480 nm ≈330/≈555 nm	Wild type and mutant *E. coli*	Measure cellular accumulation of the TMP probe and assess efflux pump activity	[88]
BODIPY‐Tyl NBD–Tyl Flu‐Tyl Alexa‐Tyl Rho‐Tyl Rho‐Des		485/535 nm 485/535 nm 492/516 nm 488/512 nm 535/580 nm 535/580 nm	*E. coli*	Study the complex formation between fluorescent macrolide derivatives and *E. coli* ribosomes via fluorescence polarization Screening the affinity of novel antibiotic derivatives and other ligands to bacterial ribosomes in the macrolide binding site	[140]
Fluoroquinolone‐derived fluorescent probes	CuAAC		*E. coli* AG102	Study bacterial penetration and efflux	[89]

One of the significant factors to consider when selecting and designing fluorescent antibiotics is the availability of the possible conjugation sites. The location where the fluorophore is attached to the antibiotics should not significantly alter its binding affinity to its target or its pharmacokinetic properties (i.e., how it is absorbed, distributed, metabolized, and excreted). Fortunately, extensive research has already been conducted to study the structure–activity relationship (SAR) of antibiotics, which consists of identifying those functional groups directly involved in the activity at the molecular level and examining to what extent they can be modified without compromising the antibiotic's effectiveness.^[^ ^38^, ^39^, ^40^, ^41^, ^42^, ^43^, ^44^ ^]^ For example, two chemical modifications near the binding site of the Van molecule (or related glycopeptide) markedly affect its antibacterial activity.^[^ ^45^, ^46^ ^]^ Accordingly, removing the crucial N‐terminal leucine eliminates the antibacterial activity of the antibiotic. Similarly, introducing a new carboxylate group by replacing the carboxamido unit of asparagine with aspartic acid reduces its antibacterial activity. Thus, structural alterations near the N terminus obstructing binding to the acyl‐D‐Ala‐D‐Ala carboxy terminus of uridine diphosphate‐*N*‐acetyl‐muramylpentapeptide impair the antibiotic's efficacy. Regarding polymyxins, several chemical and structural properties influence the antibacterial activity and LPS binding, including the amphipathic and cationic distribution of charges across the primary sequence, the lactam ring size, and the *N* ^α^ fatty acid chain.^[^ ^38^ ^]^ In particular, alterations that neutralize the positive charges of the Dab side chains often lead to a complete loss of antimicrobial activity. Expanding or reducing the cyclic ring also results in a substantial loss of antibacterial potency. Modifications to the *N* ^α^ fatty acid chain indicate that the optimal chain length is between seven and nine carbons. The unique 3D architecture of polymyxins is also required for both LPS binding and antimicrobial activity.

While SAR studies are valuable for guiding chemical modifications of antibiotics into novel compounds, such as new drugs or fluorescent derivatives, they do not always provide precise SARs. This limitation is particularly evident for antibiotics targeting bacterial internal components, as they may fail to penetrate the cells. Nevertheless, scientists often rely on SAR studies as reference points, acknowledging that the results might fall short of expectations or, in some cases, exceed them. For example, the SAR of aminoglycoside antibiotics suggests that the number and position of amino groups in the sugars attached to deoxystreptamine profoundly affect their biological activity.^[^ ^42^ ^]^ The presence of at least one amino group in these sugars is essential for maintaining biological activity, and additional substituents to deoxystreptamine–aminosugar structure, such as a ribose moiety or ribostamycin, can further enhance the biological activity of these compounds. Similarly, SAR analyses of erythromycin have identified the functional elements essential for antibacterial activity.^[^ ^47^ ^]^ These include the integrity of the lactonic macrocycle, the presence of methyl groups at positions 4, 6, 8, and 12 in their α conformation and at positions 2 and 10 in their β conformation, as well as the hydroxyl groups at positions 6, 11, and 12 in their β conformation. Removal of desosamine at position *C*5 results in complete loss of activity, whereas *L*‐cladinose has no significant impact on inducing antibacterial action. For oxazolidinones, particularly linezolid, SAR studies indicate the importance of the oxazolidinone ring, the C5—acyl aminomethyl substituent, and the N‐aryl substituent, with the 5‐acetamido methyl group in ring B playing a key role in potent antimicrobial activity.^[^ ^48^ ^]^ Meanwhile, fluoroquinolone SAR studies have described the correlation between the specific positions within the quinolone ring and their biological effects.^[^ ^49^ ^]^ For instance, inserting cyclic groups at the C‐1 position, where a nitrogen atom (N1) is present, generally enhances antimicrobial potency. The C‐3 and C‐4 positions make up the pharmacophore of the molecule. The fluorine atom at position C‐6 improves the penetrability through the bacterial wall and membrane in Gram‐positive and Gram‐negative bacteria. The C‐7 position is the most flexible site to introduce bulky molecules, which is recommended to form a carbon–nitrogen bond (C7—N) between the atoms to maintain the antibiotic effect. In the case of trimethoprim, modifications of the 2,4,5‐trimethoxybenzyl group at the 5‐position or the methyl group at the 4‐position on the pyrimidine ring make an impact on its antibacterial activity and binding affinity to bacterial enzymes. Replacing the 4‐amino group of the pyrimidine ring results in poor or no antibacterial action, indicating the need to maintain the 4‐NH_2_ group.^[^ ^50^ ^]^

In general, chemical modifications of other functional groups distant from the specific binding sites of antibiotics do not significantly alter the antibacterial activity. In contrast, appropriate adjustments to such groups may even enhance their property potency. Thus, conjugation must be carefully tailored to maintain or avoid disrupting the functional groups responsible for the antibiotic's primary activity to yield the desired fluorescent outcome. One practical approach is incorporating fluorophores with similar functional groups or intrinsic antibacterial properties into the new conjugates to compensate for the loss of activity. This work could be supported with the help of predictive tools such as molecular dynamic simulations or quantitative SAR analyses, as demonstrated during the invention of novel antibiotics in the past.

The classification of antibiotics based on their targeted components in the bacterial structure, mode of action, and treatment of certain diseases and infections provides a framework for the rational design of fluorescent antibiotics (Figure 1). To study bacterial regulation and behavior, researchers have leveraged the unique functionality of internally targeted antibiotics, which interact with bacterial ribosomes and nucleic acid (Table 4). In contrast, Van and polymyxins have been employed to investigate cell wall and membrane formation. These externally targeted antibiotics enable rapid bacterial detection and imaging, as attaching to the membrane takes a shorter time than penetrating internal components. These examples illustrate how rational engineering of antibiotics leverages bacterial structural vulnerabilities, providing options to create fluorescent toolboxes for bacterial behavior studies, detection, and treatment.

The broad‐spectrum action of antibiotics often indiscriminately affects both harmful and beneficial bacteria.^[^ ^51^ ^]^ Furthermore, many antibiotics are ineffective against MDR bacterial infections.^[^ ^52^ ^]^ As a result, the diagnostic scope is often limited to distinguishing between Gram‐positive and Gram‐negative bacteria, usually within nonresistant communities, using fluorophore‐conjugated antibiotics, with a few exceptions discussed later in the review. This has driven an increasing demand for novel antibiotics or innovative technologies. However, considering the current circumstances, the repetitive cycle of antibiotics (or any antimicrobial agents) discovery and appearance of resistance will likely persist unless research shifts toward developing ultra‐narrow‐spectrum antibiotics, guided by the growing knowledge of infection‐specific bacterial profiles. Owing to the power of RNA‐guided destruction of nucleic acids, clustered regularly interspaced short palindromic repeats (CRISPRs)‐Cas (CRISPR‐associated) systems have recently become a promising solution for developing next‐generation antimicrobials to address infectious diseases, particularly those caused by MDR pathogens. Moreover, the flexible programmability of CRISPR‐Cas systems allows them to selectively target specific bacterial membranes within a large population, which enables CRISPR‐Cas antimicrobials to modulate the composition of a complex bacterial population precisely. This targeted approach makes CRISPR‐Cas antimicrobials particularly effective in treating infections in natural consortia, surpassing conventional antibiotics in specificity and control.^[^ ^53^, ^54^ ^]^ These systems could also help reverse antibiotic resistance before detecting and eliminating resistant bacteria. Similarly, other emerging technologies such as bioinformatics, SAR, phage therapy, zinc finger nuclease, and peptide nucleic acid hold significant potential in combating the challenges posed by antibiotic resistance.^[^ ^55^ ^]^ With rapid technological advancements, numerous novel antimicrobial agents remain to be discovered across the bacterial kingdom.^[^ ^2^ ^]^ However, despite the substantial future potential, limitations in current technologies, financial viability, and many other aspects continue to impede progress. Addressing these issues requires global awareness, sustained funding, cross‐disciplinary collaboration, and the collective commitment of scientists, clinicians, and policymakers.

### 2.2 Selection of Fluorophores

Selecting the appropriate fluorophores is just as crucial as identifying the suitable targeting element for accurate diagnosis, as it significantly impacts the effectiveness, sensitivity, and specificity of fluorescent labeling. An ideal fluorophore should exhibit high brightness and photostability to ensure precise and long‐lasting imaging. Key factors to consider include size, lipophilicity, and electrostatic charge: smaller fluorophores minimize steric hindrance. In contrast, positive charges are less likely to be repelled by the negatively charged surfaces of bacteria.^[^ ^56^ ^]^

**Figure 2:**
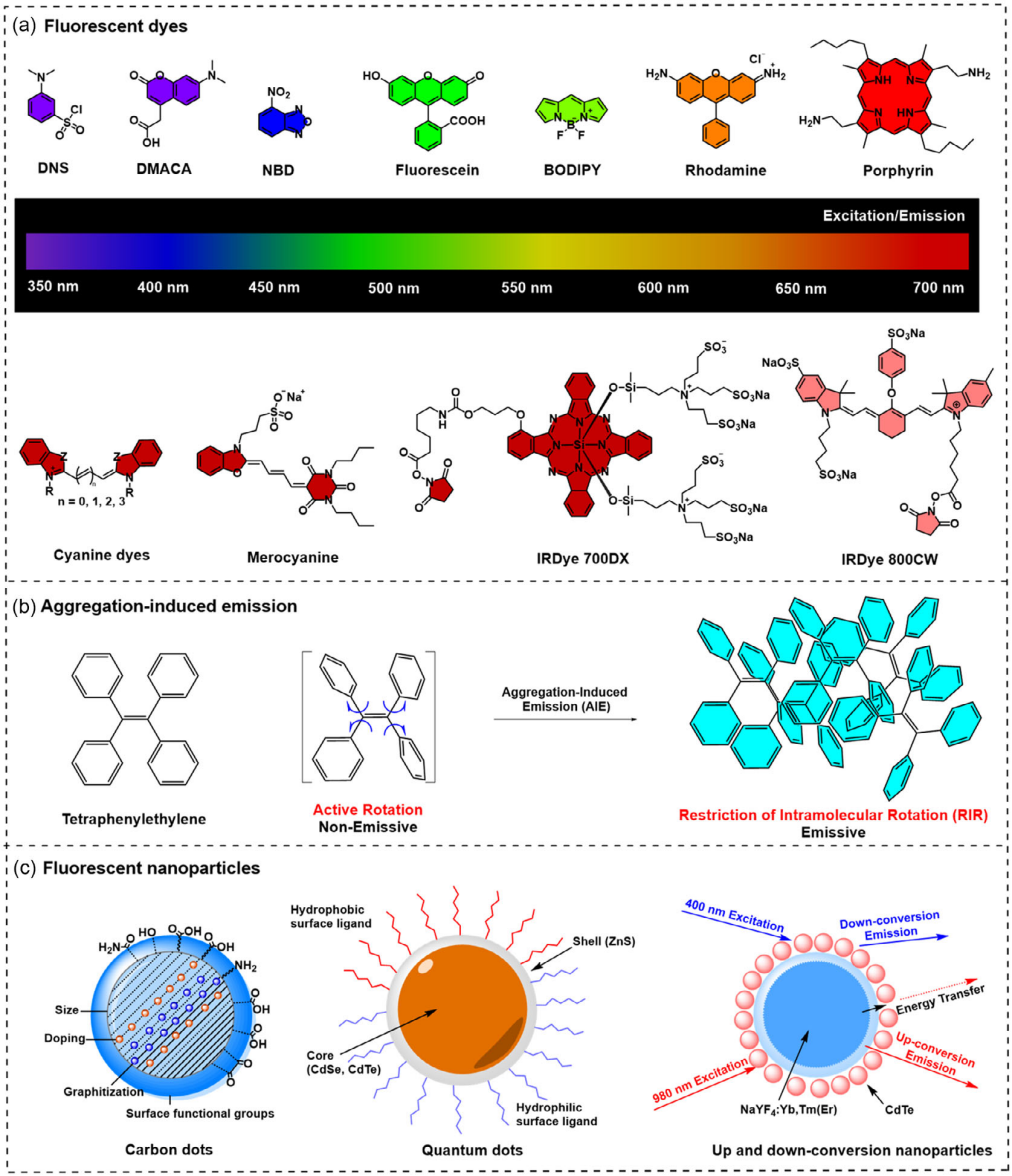
Potential fluorophores incorporated with antibiotics to develop fluorescent antibiotics. a) Chemical structures of conventional fluorescent dyes with excitation/emission wavelengths ranging from visible to infrared spectrum. b) Chemical structures and fluorescent mechanism of tetraphenylethylene (TPE), representing AIE dyes. c) Chemical structures of several fluorescent nanoparticles discussed in this review.

Traditional fluorescent dyes are commonly used due to their bright fluorescence and a broad spectrum of excitation/emission wavelengths ranging from visible to infrared spectrum (**Figure** **2**). However, these compounds suffer from poor photostability and rapid photobleaching, limiting their utility in long‐term imaging applications.^[^ ^57^ ^]^ In contrast, aggregation‐induced emission (AIE) represents a unique class of fluorophore that exhibits enhanced fluorescence upon aggregation, overcoming the aggregation‐caused quenching (ACQ) observed in conventional dyes. This property makes AIE powerful tools for applications such as fluorescence sensors and biological probes.^[^ ^58^, ^59^ ^]^

Quantum dots (QDs) provide high quantum yield, narrow and tunable emission spectra, and excellent photostability, but toxicity concerns and instability in biological matrices hinder their use.^[^ ^60^ ^]^ Carbon dots (CDs) offer properties similar to QDs, with generally fewer toxicity concerns. However, the variations in their size, quantum yields, and broad emission spectra limit their use in applications requiring precise color purity.^[^ ^61^, ^62^ ^]^

While down‐conversion nanoparticles (DCNPs) require UV–visible light for excitation and can potentially damage cells and tissues, up‐conversion nanoparticles (UCNPs), such as NaYF4:Yb, Tm, and Fe derivatives, absorb low‐energy light and emit at higher energy. This allows deep tissue penetration and minimal photodamage, making them particularly suitable for in vivo applications.^[^ ^63^, ^64^ ^]^

The specific demands of the application, including the type of bacteria, the imaging environment, and the desired resolution and sensitivity, guide the choice of fluorophore. Since each fluorophore comes with its advantages and disadvantages,^[^ ^65^ ^]^ the careful selection and optimization of suitable fluorophores is vital for achieving reliable and accurate bacterial detection and imaging.

### 2.3 Fluorescent Designs for Fluorescent Antibiotics

The mechanisms of fluorescence emission and quenching of fluorophores immensely influence the design of fluorescent probes to achieve desired properties such as enhanced brightness, improved photostability, or sensitivity to environmental factors. Grasping this fundamental knowledge facilitates the creation of sophisticated sensing systems that exploit fluorescence and quenching phenomena across various applications, ranging from cellular imaging and biomolecular interaction studies to environmental monitoring and medical diagnostics. It also helps optimize environmental conditions, ensuring that fluorescent probes are used to their full potential and pushing the boundaries of possibilities in molecular detection, imaging, and sensing applications.

Fluorescence occurs at the molecular level when a molecule absorbs and then emits light, which happens very quickly, usually within nanoseconds. Thereby, fluorescence ceases almost immediately when the excitation light source is removed. The probability of a molecule fluorescing is described by its quantum yield, which is the ratio of emitted to absorbed photons. The molecular structure and the chemical environment largely govern the intensity of fluorescence.^[^ ^66^, ^67^ ^]^ For instance, the most intense fluorescence is found in compounds containing aromatic groups with low‐energy *π* ‐> *π* ^*^ transitions. A few aliphatic, alicyclic carbonyl, and highly conjugated double‐bond structures also exhibit fluorescence. Most unsubstituted aromatic hydrocarbons fluoresce in solution, with the quantum efficiency increasing as the number of rings and the degree of condensation extends. Fluorescence is particularly favored in molecules with rigid structures, which explains the increased fluorescence of organic chelating agents when complexed with a metal ion. The quantum efficiency of a fluorophore is reduced with increasing temperature and when its solvent contains heavy atoms such as carbon tetrabromide and ethyl iodide or when heavy atoms are substituted into the fluorescing compound. The fluorescence of aromatic compounds with basic or acid substituent rings is usually pH dependent, with the emission changes arising from different resonance structures associated with the acidic and basic forms of the molecule.

For over two decades, the discovery of AIE molecules has challenged conventional principles of molecular fluorescence.^[^ ^58^, ^68^ ^]^ The unique behavior of these compounds stems from their distinctive molecular design, usually a propeller‐like structure with rotatable groups, such as those found in tetraphenylethylene (TPE) or hexaphenylsilole (Figure 2b). In dilute solutions, these molecules undergo rapid intramolecular rotations and vibrations, which provide non‐radiative pathways for excited‐state energy dissipation, resulting in weak or no fluorescence. However, when AIE molecules aggregate, their intramolecular motions become restricted due to spatial constraints, a phenomenon known as restriction of intramolecular rotations or, more broadly, restriction of intramolecular motion. This restriction blocks non‐radiative decay pathways and results in the emission of strong fluorescence. Moreover, the twisted structure of AIE molecules prevents strong π–π stacking interactions in the aggregated state, further avoiding the ACQ effect.

Fluorescence quenchers can also be paired with fluorophores to exploit different fluorescent mechanisms in fluorescent antibiotics. These quenchers work by reducing or eliminating the fluorescence emission of a fluorophore via various mechanisms. These mechanisms include collisional (dynamic) quenching, where the quencher collides with the excited fluorophore, causing it to return to its ground state without emitting a photon; static quenching, which involves the formation of a nonfluorescent complex between the fluorophore and quencher in the ground state;^[^ ^69^ ^]^ Förster resonance energy transfer (FRET), where energy is non‐radiatively transferred from an excited donor fluorophore to an acceptor quencher through long‐range dipole–dipole interactions;^[^ ^70^ ^]^ Dexter electron transfer, a short‐range phenomenon involving electron exchange between the fluorophore and quencher;^[^ ^71^ ^]^ photoinduced electron transfer, which occurs when an electron is transferred from a donor to an acceptor nearby;^[^ ^72^ ^]^ and internal conversion and vibrational relaxation, where quenchers enhance non‐radiative pathways, such as the chemical properties of the fluorophore and quencher, their relative concentrations, the distance between them, and the surrounding environment. As quenchers are attached to fluorescent probes, the resulting conjugates become weakly emissive. However, in the presence of bacteria or stimulated factors such as pH or temperature changes, enzymes, and reactive oxygen species (ROS), these quenchers are cleaved, increasing fluorescent intensity.^[^ ^73^ ^]^ In contrast, some fluorescent probes were designed to bring the quencher and fluorophore in close proximity to trigger FRET phenomena upon the addition of bacterial targets, allowing the measurement of fluorescent quenching accordingly.^[^ ^74^ ^]^

Leveraging the innovative legacy of fluorescent probes, scientists can develop antibiotic derivatives with transformative fluorescent activation functions, such as ON, ON–OFF, ON–OFF–ON, and OFF–ON configurations. The ON mechanism features constant fluorescence emission, which is ideal for tracking molecule distribution but is potentially affected by high background signals. ON–OFF probes start with fluorescence that quenches upon target binding, often due to electronic structure changes, making them useful for detecting specific molecules or ions.^[^ ^75^, ^76^ ^]^ ON–OFF–ON probes undergo three stages: initial fluorescence, quenching upon target binding, and fluorescence recovery. These stages are suitable for detecting multistep processes, such as antibiotic uptake and localization within bacterial cells.^[^ ^77^, ^78^, ^79^ ^]^ In contrast, OFF–ON probes activate fluorescence upon target binding or enzymatic processing, offering high sensitivity due to low background signals.^[^ ^80^, ^81^, ^82^ ^]^

Smart fluorescent probes represent a promising frontier in antibiotic research, offering precise, real‐time tracking of bacterial infections. These probes, designed to remain inactive until encountering target bacteria, could enable highly selective detection with minimal background interference. Their functionality relies on tailored molecule modifications, such as integrating fluorophores with stimuli‐responsive chemical groups such as enzyme sensitivity, pH responsiveness, and redox‐controlled fluorescence, which trigger fluorescence upon interaction with bacteria. Among the various configurations, OFF–ON and ON–OFF–ON most accurately embody the concept of smart fluorescent probes. Beyond bacterial detection, smart probes could revolutionize drug delivery systems, ensuring therapeutics are activated only upon reaching their intended targets. However, the current smart fluorescent probes remain primarily theoretical, with challenges in optimizing probe stability, selectivity, and signal‐to‐noise ratios (SNRs). One major limitation is stability in physiological conditions, as some probes degrade prematurely due to environmental factors or metabolic interactions. Moreover, ensuring highly specific activation solely upon bacterial presence requires fine‐tuning of the probes chemical properties to avoid unintended fluorescence caused by unrelated stimuli. The ability of these probes to penetrate deeper tissue while maintaining a strong fluorescence signal also remains a hurdle for clinical applications.

By applying these advanced fluorescent functions, researchers could engineer precise, adaptable tools that significantly enhance bacterial detection and analysis efficiency and accuracy. Such technologies can potentially transform the landscape of medical diagnostics and microbial research.

### 2.4 Chemical Strategies for the Synthesis of Fluorescent Antibiotics

**Figure 3:**
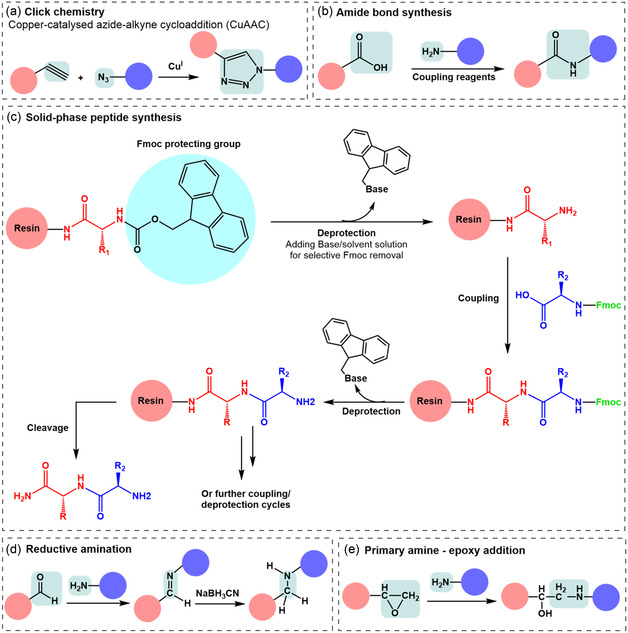
Types of chemical conjugation/synthetic pathway for fluorescent antibiotics. a) Orthogonal click reactions: copper‐catalyzed azide‐alkyne cycloaddition (CuAAC) reaction. b) Chemical structure of amides and the conventional chemical method for amide bond synthesis. Common coupling reagents include EDC, 1‐ethyl‐3‐(3‐dimethylaminopropyl)carbodiimide; CDI, carbonyldiimidazole; DCC, dicyclohexylcarbodiimide; T3P, n‐propylphosphonic acid anhydride; HATU, (1‐(bis(dimethylamino)methylene)‐1H‐1,2,3‐triazolo(4,5‐b)pyridinium 3‐oxide hexafluorophosphate; HBTU, N,N,N′,N′‐tetramethyl‐*O*‐(1H‐benzotriazol‐1‐yl)uronium hexafluorophosphate. c) General scheme of solid‐phase peptide synthesis (SPPS) steps. Protecting groups (PGs) will be on the amine nitrogen for iterative deprotection and any potentially reactive side chain groups. d) The reductive amination reaction involves two steps to convert the carbonyl compound into an amine. Step 1: formation of an intermediate imine. Step 2: reduction of the imine using metal hydrides like sodium borohydride (NaBH_4_) or sodium cyanoborohydride (NaBH_3_CN) as reducing agents. e) Epoxy‐amine reaction scheme.

Various reactions can synthesize fluorescent‐based antibiotics, including click chemistry, amide bond formation, solid‐phase peptide synthesis (SPPS), reductive amination, epoxy‐amine reaction, and electrostatic interaction (**Figure** **3** and Table 1, 2, 3, 4). These methods can be employed individually or in combination. Following conjugation, the properties of new conjugates are expected to change somewhat due to the change in chemical compositions, the loss of functional groups, and the exposure or hindrance of conjugated molecules. The following sections will explore the available synthetic routes to produce fluorescent antibiotics and discuss the capacity of the modified compounds to retain the same activity as their parent counterparts.

#### 2.4.1 Click Chemistry

Initially coined by K. Barry Sharpless, click chemistry refers to a set of highly efficient, selective, and versatile reactions that rapidly join molecule entities.^[^ ^83^ ^]^ One of the most well‐known reactions in this category is the 1,3‐dipolar cycloaddition of azide and alkyne to form 1,2,3‐triazole, known for its high yield and simplicity, and due to the irreversible and quantitative nature of triazole formation (Figure 3a). This reaction has been widely applied in various fields, including macromolecules and surface chemistry, biological applications such as labeling large molecules and constructing supermolecule structures, chemical synthesis, materials science, and drug development.^[^ ^84^, ^85^, ^86^ ^]^

In 2014, a research team led by Mark A. T. Blaskovich and Matthew A. Cooper developed an azide‐functionalized version of the oxazolidinone antibiotic linezolid. They subsequently linked this analog to two different alkyne‐functionalized fluorophores, 7‐(dimethylamino)‐coumarin‐4‐acetic acid (DMACA) and 7‐nitrobenzofurazan (NBD), to create fluorescent probes in the form of triazole‐linked conjugates.^[^ ^87^ ^]^ In a subsequent study, they employed the same strategy to prepare fluorescent derivatives of trimethoprim, adding a fluorophore called alkyne‐functionalized dansyl (DNS, dimethylaminosulfonyl).^[^ ^88^ ^]^ More recently, they have expanded their efforts by developing fluorescent probes derived from the fluoroquinolone antibiotic ciprofloxacin.^[^ ^89^ ^]^

These synthetic procedures included multiple steps: preparing antibiotics–azide from antibiotics itself via alkylation with tosylated 3‐azidopropanol, followed by CuAAC coupling with NBD–, DMACA–, and DNS–alkynes. These antibiotic fluorescent derivatives had reduced antimicrobial activity, while the retention of fluorescent activity of these conjugates compared to the original dyes was not reported. The antimicrobial efficacy of the linezolid–DMACA probe was reduced, exhibiting a minimum inhibitory concentration (MIC) greater than 64 μg mL^−1^. In contrast, the linezolid–NBD probe maintained partial activity, with MICs ranging from 4 to 32 μg mL^−1^. All trimethoprim (TMP)‐based fluorescent probes demonstrated a loss of antimicrobial effectiveness, with MICs greater than 64 μg mL^−1^. However, these probes regained their potency against the *E. coli* ΔtolC mutant, with MICs between 0.25 and 8 μg mL^−1^. The cipro–DMACA probe's activity was consistently lower across various bacterial strains, ≈50 times relative to ciprofloxacin. The cipro‐NBD probe displayed a similar trend to the cipro–DMACA probe when tested against wild‐type strains. Still, its activity varied significantly against *E. coli* mutants, exhibiting up to a 100‐fold increase in activity.

In their most recent study, Blaskovich and colleagues synthesized azido‐Van containing different linker properties in length and hydrophobicity, Van‐C8‐N3 and Van‐polyethylene glycol (PEG)3‐N3. These were then linked to alkyne‐derivatized NBD and DMACA via a CuAAC reaction to form Van‐C8‐NBD, Van‐C8‐DMACA, and Van‐C3PEG‐NBD.^[^ ^56^ ^]^ Adjustments in reaction conditions were required since Van is known to chelate copper.^[^ ^90^ ^]^ The fluorescent probes Van‐C8‐NBD and Van‐C8‐DMACA demonstrated enhanced antibacterial activity against multiple strains, roughly two to four times greater than the parent Van, likely due to lipophilicity. Van‐C3PEG‐NBD, with the PEG3 linker, maintained antimicrobial activity comparable to Van. Moreover, Van‐C8‐NBD showed twice the activity against all tested *Staphylococcus aureus* (*S. aureus*) strains compared to commercially available fluorescent probes vancomycin‐ conjugated boron dypyrromethene (Van–BODIPY,V34850, Invitrogen), whereas vancomycin‐conjugated fluorescein isothiocyanate (Van–FITC, SBR00028, Sigma‐Aldrich) did not display antimicrobial activity at the highest concentration tested (32 μg mL^−1^).

A light‐up probe with AIE properties was created through a “click” reaction.^[^ ^91^ ^]^ Van was functionalized with propargylamine using 1‐ethyl‐3‐(3‐dimethylaminopropyl)carbodiimide (EDC)/N‐hydroxysuccinimidyl ester (NHS) coupling to create an alkyne–Van. This compound was subsequently linked to an AIE fluorogen derived from TPE modified with 3‐Azidopropyl tosylate using CuACC reaction.^[^ ^92^ ^]^ The study demonstrated that AIE‐2Van and Van have comparable inhibitory effects on *Bacillus subtilis* (*B. subtilis*), but their efficacy drops against Van‐resistant *Enterococcus* (VRE) strains, with AIE‐2Van being less affected. In a recent study, AIE‐DCM‐2polymyxinB was formulated by attaching an AIEgen AIE‐DCM to two Gram‐negative bacterium‐targeting peptides of polymyxin B (PMB).^[^ ^93^ ^]^ The AIE‐DCM core was prepared with modifications from the previous work of the same team^[^ ^94^ ^]^ to introduce azide functional groups. These were then linked to Mal‐Pra—a molecule with a maleimide and an alkyne group—via a CuAAC click reaction. The resulting intermediate, AIE‐DCM‐2Mal, was further conjugated with a thiol group on the backbone of PMB through a thiol‐maleimide Michael addition reaction, forming the final product AIE‐DCM‐2polymyxinB. However, the team did not report on how the properties of the probe may differ from the parent antibiotics or the original AIE compound.

A methodology for synthesizing PMB‐modified conjugated oligomer nanoparticles (PMB–CONs) devoid of copper or any other catalyst was reported.^[^ ^95^ ^]^ The process entailed a click chemistry reaction between azide‐modified CONs and dibenzocyclooctyne (DBCO)‐functionalized PMB. CON was first prepared by reprecipitating BFTB and DSPE‐PEG2000‐N3, likely due to lipid–lipid bilayer formation. PMP was then incubated with DBCO–NHS to undergo an NHS ester‐amine reaction, forming PMP–DBCO. This intermediate was further incubated with the azide nanoparticle CON, resulting in the successful coupling of PMP to the surface of CON, as confirmed by zeta potential characterization. A comparison between the properties of PMP–CON and those of the precursor materials was not reported.

In contrast, bioorthogonal chemistry is a specialized subset built upon the principles of click chemistry. It is an approach in which a biomolecule (a cell wall component, an amino acid, a nucleotide, or a metabolite) is labeled with a small chemical group, or “handle,” that does not interfere with their biological function (the molecule is therefore bioorthogonal). These handles can then be linked to detectable tags, such as fluorescent markers, which allow visualization, quantification, or selective isolation of cellular processes using the original labeled molecules. These bioorthogonal reactions, including Staudinger ligation reaction, copper‐catalyzed azide‐alkyne cycloaddition (CuAAC), strain‐promoted alkyne‐azide cycloaddition, photoclick reactions, strain‐promoted alkyne‐nitrone cycloaddition (SPANC), and inverse‐electron‐demand Diels–Alder reaction, have been extensively employed in the development of fluorescent probes.^[^ ^96^, ^97^, ^98^ ^]^ However, only a few studies applied bioorthogonal chemistry to designing fluorescent antibiotics to detect bacteria.

Chung et al. described a two‐step labeling method for Gram‐positive bacteria using antibiotics (Van or daptomycin derivatives) in conjunction with magnetofluorescent nanoparticles (MFNPs).^[^ ^99^ ^]^ The approach used *trans*‐cyclooctene (TCO) derivatives of these antibiotics as the targeting ligands. Following the bioorthogonal cycloaddition reaction with tetrazine‐modified MFNPs (MFNPs–Tz), bacteria were labeled and can be detected via fluorescent or magnetic sensors. TCO‐antibiotic derivatives were prepared by modifying the primary amine groups of the antibiotics with TCO‐NHS. At the same time, amine‐reactive tetrazine (Tz‐NHS) was synthesized separately and reacted with the amine‐functionalized MFNPs to create MFNP–Tz. A study led by Pezacki's group demonstrated the functionalization of Van with an endocyclic nitrone for cell‐surface fluorescent labeling of bacteria using SPANC chemistry.^[^ ^100^ ^]^ Modifications of Van were made possible due to the presence of two potential and mutually exclusive sites that are unlikely to interfere with its tertiary structure. A primary amine on the vancosamine sugar serves as a suitable site for coupling reactions with activated carboxylic groups. In contrast, only one carboxylic is exposed, as the rest of the amino acid functional groups are part of the peptide bonds. Modifying Van at this free carboxylic acid causes the least perturbation of its function. Hence, the nitrone was attached to Van at its free carboxylic acid position using a polyethylene glycol (PEG) spacer to ensure accessibility to strained alkynes. The study confirmed that nitrone‐functionalized Van efficiently labels bacterial PG, with experiments in *Lactococcus lactis* revealing strong fluorescence even at low concentrations (20 mm) after 30 min of incubation.

Despite significant advances, click‐chemistry‐based approaches are not without their limitations. The first is the toxicity stemming from some of the reagents used in specific bioorthogonal ligation reactions. For instance, the widely used copper‐catalyzed Huisgen ligation relies on a Cu(I) catalyst, which is toxic to most cells. As a result, its application is restricted to scenarios where bacteria are either fixed before labeling or analyzed shortly after live‐cell labeling.^[^ ^101^ ^]^ Furthermore, the stability of bioorthogonal reagents can be compromised in biological samples due to interactions with cellular components or oxidative bursts.^[^ ^102^, ^103^ ^]^ Differential label incorporation across species and competition with natural biomolecules can also limit efficiency in multi‐species communities. Some ligation reactions are not entirely bioorthogonal, causing off‐target reactions and reducing specificity in complex biological systems. Lastly, unlike traditional laboratory reactions, bioorthogonal reactions are designed not for bulk synthesis but for precise chemical modifications within complex biological systems.^[^ ^104^ ^]^

#### 2.4.2 Amide Bond Synthesis

Amide bond formation accounted for a considerable part of molecular conjugations, where the role of coupling reagents is critical (Figure 3b). Common coupling reagents include EDC, (1‐(bis(dimethylamino)methylene)‐1H‐1,2,3‐triazolo(4,5‐b)pyridinium 3‐oxide hexafluorophosphate (HATU), N,N,N′,N′‐tetramethyl‐*O*‐(1H‐benzotriazol‐1‐yl)uronium hexafluorophosphate (HBTU), T3P, and CDI. These reagents activate the carboxylic acid, forming an activated ester or acylating agent, which then reacts with amine or ammonia to create the amide bond. Each reagent offers unique benefits (e.g., reaction efficiency and ease of purification) and specific challenges depending on the reaction conditions (e.g., reaction times, solvents compatibility, temperature sensitivity, and potential for side reactions or racemization), reflecting the importance of their selection for the practical synthesis of amide conjugates.^[^ ^105^, ^106^, ^107^ ^]^

The EDC/NHS coupling system, for example, has been employed to link antibiotics such as Van, polymyxins, and teicoplanin to the surface of various types of fluorescent nanoparticles, including silicon NPs (SiNPs),^[^ ^108^ ^]^ CDs,^[^ ^109^, ^110^ ^]^ UCNPs,^[^ ^111^ ^]^ tri‐doped alkaline earth sulfide NPs,^[^ ^112^ ^]^ DCNPs,^[^ ^113^ ^]^ polymer dots (Pdots).^[^ ^114^ ^]^ Similarly, fluorescent dyes modified with NHS groups have been used to create a range of fluorescent antibiotics through NHS ester‐amine reaction, resulting in Van‐IRDye800CW,^[^ ^115^ ^]^ PTPVan,^[^ ^116^ ^]^ Vanco–Cy3,^[^ ^117^ ^]^ Van–Cy5 and PMP–Cy3,^[^ ^118^ ^]^ BODIPY–erythromycin,^[^ ^119^, ^120^ ^]^ and fluorescein–neomycin.^[^ ^121^ ^]^

A divalent Van derivative was created by reacting Van with a porphyrin derivative to afford Van in carboxamide form by employing HBTU as the coupling reagent, yielding a 53.6% purified product through reversed‐phase HPLC.^[^ ^122^ ^]^ Van–porphyrin conjugates demonstrated potent MIC activity against *B. subtilis*, 3 and 2 μm for monovalent and divalent Van derivatives, respectively, comparable to the parent Van molecule. However, these compounds showed a distinct decrease in antimicrobial activities against VRE strains (>15 μm), suggesting that while enhanced affinities for bacterial cell wall precursors were observed, this did not directly correlate with potent MIC activity against VRE.

The conjugation of IRdye800CW and IRdye700DX to Van and amphotericin B was described utilizing coupling agents such as HBTU or HATU, as a continuation of a previous work on near‐infrared probe Van‐800CW.^[^ ^123^ ^]^ The process resulted in conversion rates above 50%. The synthesized Van‐IRdye800CW retains high selectivity for binding Gram‐positive bacteria, as demonstrated in in vitro experiments. However, the conjugation to IRdye800CW and IRdye700DX, which bind to the secondary amine at the N‐terminus of Van, showed a higher MIC value against *Staphylococcus epidermidis* (*S. epidermidis*). This decrease in antibacterial potency is likely due to a reduced affinity for the D‐Ala‐D‐Ala dipeptide in the bacterial cell wall, which is the target of Van.

#### 2.4.3 Solid‐Phase Peptide Synthesis

9‐fluorenylmethyloxycarbonyl (Fmoc) SPPS (Fmoc‐SPPS), a method pioneered by Robert Bruce Merrifield in the 1960s, is a mainstay in peptide synthesis, enabling the efficient and stepwise construction of peptides (Figure 3c). The process begins with the attachment of the first amino acid to a solid resin, protected at the N‐terminus by the Fmoc group, which is selectively removed with a base to expose the free amine for the following coupling reaction. Subsequent amino acids, also Fmoc‐protected, are added sequentially, with side chains protected to prevent side reactions. The process includes repeated cycles of deprotection and coupling, followed by washing to remove excess reagents. After the peptide chain is fully assembled, it is cleaved from the resin and the side chain protecting groups are removed, typically with trifluoroacetic acid. The final peptide is then purified and characterized to confirm its structure and purity. This method simplifies the synthesis process by facilitating the removal of excess reagents and byproducts through filtration and washing.^[^ ^124^, ^125^ ^]^

A fully synthetic fluorescent probe, [dansyl‐Lys]^1^polymyxin B3 (DPmB3), was developed using Fmoc‐SPPS.^[^ ^126^ ^]^ The goal was to create a dansylated PMB structure with minimal substitution, as they found a complex mixture of mono‐, di‐, tri‐, and tetra‐dansyl‐substituted PMB in semisynthetic and commercial products. The synthesized probe, DPmB3, had an affinity for LPS comparable to PMB and colistin but lacked antibacterial activity, with an MIC greater than 16 μg mL^−1^. Subsequently, the same team designed a polymyxin probe that preserved the pharmacological properties of PMB.^[^ ^127^ ^]^ The design strategy employed the regioselective incorporation of the dansyl group into the hydrophobic N‐terminal center of the PMB core scaffold, which has a minimal impact on the native antibacterial activity of PMB. The probe demonstrated antibacterial activity against certain strains of *Pseudomonas aeruginosa* (*P. aeruginosa*), *Acinetobacter baumannii* (*A. baumannii*), and *Klebsiella pneumoniae* (*K. pneumoniae*) that was similar to that of PMB and colistin and showed some activity against polymyxin‐resistant strains of *P. aeruginosa* and *A. baumannii*.

Continuing their research, the team rationally designed fluorescent polymyxin probes focusing on incorporating a dansyl fluorophore at the hydrophobic center of the polymyxin core structure.^[^ ^128^ ^]^ This led to the creation of four novel monodansylated PMB probes (MIPS‐9541, MIPS‐9542, MIPS‐9543, and MIPS‐9544). Among these, MIPS‐9541, which had a dansyl–octylglycine group replacing the N‐terminal fatty acyl chain, showed slightly lower activity (MICs 4‐8 μg mL^−1^) compared to the other probes (MICs 1‐4 μg mL^−1^). The probes having the dansyl fluorophore at the N terminus (MIPS‐9542) or positions 6 (MIPS‐9543) or 7 (MIPS‐9544) of the polymyxin core maintained better antimicrobial activity, indicating that the position of the dansyl group affects activity. The substitution at the N terminus with the dansyl fluorophore and a C8 fatty acyl chain (MIPS‐9541) was less favorable for retaining antimicrobial activity, possibly due to steric effects or conformational changes that hinder optimal binding to lipid A.

Two Gram‐negative specific fluorescent probes, PMS‐DNS and PMS‐Cy‐NO_2_, were developed using similar SPPS.^[^ ^129^ ^]^ The PMS‐DNS demonstrated potent antibacterial activity against *Escherichia coli* (*E. coli*) (1 μg mL^−1^) and various (ESKAPE) pathogens *enterococcus faecium, staphylococcus aureus, klebsiella pneumoniae, acinetobacter baumannii, pseudomonas aeruginosa, and enterobacter species*, which are significant culprits in nosocomial infections, including Gram‐negative species like *K. pneumoniae* (4 μg mL^−1^), *A. baumannii* (0.5 μg mL^−1^), *P. aeruginosa* (16 μg mL^−1^), and *Enterobacter cloacae* (*E. cloacae*) (1 μg mL^−1^). However, PMS‐DNS exhibited no bactericidal activity against Gram‐positive pathogens, such as *Enterococcus faecium* (*E. faecium*) and *S. aureus*, with MIC values greater than 128 μg mL^−1^. PMS‐Cy‐NO_2_ showed slightly less antibacterial activity compared to PMS‐DNS, with MICs of 8, 4, 16, and 4 μg mL^−1^ against *K. pneumoniae*, *A. baumannii*, *P. aeruginosa*, and *E. cloacae*, respectively. Each probe's fluorescence emission was intense, with an emission peak around 550 nm for PMS‐DNS and 810 nm for PMS‐Cy‐NO_2_.

These findings highlight the versatility of Fmoc‐SPPS in producing fluorescent probes with tailored properties for bacterial detection and antimicrobial research. SPPS offers numerous advantages over solution synthesis,^[^ ^125^, ^130^ ^]^ such as carrying out all reactions in a single vessel, employing excess amino acid and reagents to drive reactions to completion and subsequently filtering out these components without the need for intermediates purification after each step, as well as its automation capability due to the repetitive nature. However, SPPS also faces some serious limitations.^[^ ^131^, ^132^ ^]^ A notable one is that removal of by‐products during synthesis is impossible. Racemization poses another challenge, where specific amino acids, like cysteine and histidine, may undergo unwanted stereochemical changes during coupling reactions, compromising the integrity of the final products. The process also requires excessive reagents, leading to higher costs and raising environmental concerns due to waste generation. Furthermore, SPPS can struggle with secondary structure formation, where β‐sheet or α‐helical structures within peptides can interfere with synthesis, leading to incomplete sequences or reduced yields. Finally, while automation has enhanced efficiency, SPPS still requires meticulous optimization of reaction conditions, including solvent choice and coupling strategies, to achieve high yield and purity.

#### 2.4.4 Reductive Amination

Reductive amination is a fundamental synthetic approach that forms an imine or enamine intermediate by an aldehyde or ketone reaction with an amine (Figure 3d). In particular, the amine acts as a nucleophile, attacking the carbonyl carbon of the aldehyde or ketone to form a carbinolamine intermediate, which then undergoes dehydration to produce iminium ions. Subsequent reduction of this intermediate, often using a reducing agent such as sodium cyanoborohydride (NaBH_3_CN) or sodium borohydride (NaBH_4_), yields the desired amine product. This method is highly valued for its specificity, efficiency, and ability to be performed in a one‐pot procedure, minimizing waste and simplifying purification processes.^[^ ^133^, ^134^ ^]^

The Merocy–Van compound was reportedly produced by the reductive amination of amine‐terminated Van and aldehyde‐containing merocyanine dyes.^[^ ^135^ ^]^ This compound inhibited the growth of *S. aureus* and *Streptococcus pneumoniae* (*S. pneumoniae*) at low concentrations (1 μm) without affecting *E. coli*. Additionally, it retained nontoxicity and nonhemolytic properties toward eukaryotic cells, highlighting its potential for clinical application.

Following this work, another group developed two innovative merocyanine dyes with carboxylic acid groups designed to facilitate the linkage of PMB using HSPyU, resulting in redshifted imaging probes.^[^ ^136^ ^]^ However, comprehensive evaluations of these probes, similar to those performed on the Van derivative probes, have yet to be reported. The same group also introduced a probe based on the reductive amination of Van with a 4‐nitrobenzoxadiazole (NBD)‐based aldehyde.^[^ ^137^ ^]^ The probe exhibited antibacterial activity against *S. aureus* with an MIC concentration of 4 μm, 16 times higher than Van (<0.25 μm). As expected, Van–NBD and Van showed no antibacterial activity against the Gram‐negative bacteria tested (MIC > 64 μm).

These advancements underline the potential of reductive amination for designing functionalized antibiotic probes, paving the way for novel imaging and antibacterial applications.

#### 2.4.5 Amine‐Epoxy Addition Reaction

By employing an epoxy‐amine reaction that entails a solitary hydrothermal treatment of 3‐glycidoxypropyltrimethoxysilane and rose bengal, Fu‐Gen Wu et al. successfully synthesized ultrasmall epoxy group‐functionalized organosilica nanodots (OSiNDs) characterized by an ≈31% quantum yield of photoluminescence.^[^^138^^]^ These OSiNDs could be conjugated with Van by stirring the mixtures at an elevated temperature (e.g., 60 °C) for several hours without activating reagents. The reaction likely occurred between the amine groups of Van molecules and unreacted oxirane groups of OSiNDs. The successful conjugation of Van to OSiNDs (OSiNDs–Van) was confirmed by zeta potential characterization. The OSiNDs–Van successfully inhibited the growth of MDR bacteria and even eliminated their biofilms, demonstrating improved efficacy compared to Van alone. This enhancement is attributed to the multifunctional capabilities of the OSiNDs–Van, which not only retains the antimicrobial properties of Van but also leverages the photoluminescence and biofilm penetration abilities of the OSiNDs.

## 3 Applications for Fluorescent Antibiotics

### 3.1 Studying the Intricate Nature of Bacteria

**Figure 4:**
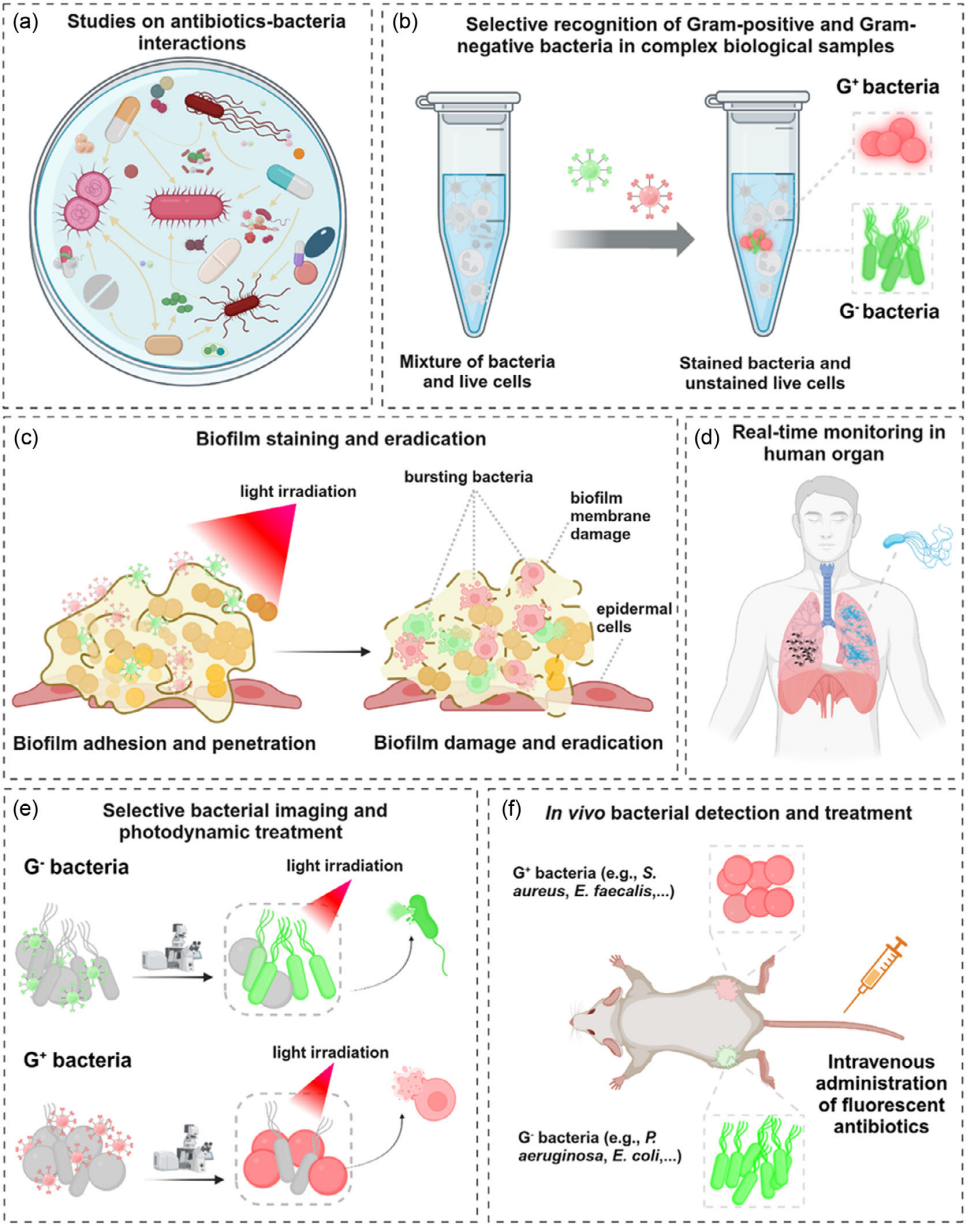
Potential biomedical applications of fluorescent antibiotics. a) Studies on antibiotic‐bacteria interactions for drug development and resistance mechanism discovery. b) Selective recognition of Gram‐positive and Gram‐negative bacteria in complex biological samples. c) Biofilm staining and eradication. d) Real‐time monitoring of infections in human organs. e) The selective bacterial imaging and photodynamic treatment. f) In vivo bacterial detection and treatment.

Understanding the complex interactions between antibiotics and bacteria provides valuable knowledge on the mechanisms of antibiotic actions at a molecular level and the evolution of bacterial resistance, which enables researchers to devise new antibacterial compounds and approaches to combat antibiotic‐resistant bacteria. Progress in fluorescent technologies and the development of fluorescent antibiotics have underpinned the deeper exploration of these interactions and the intricate nature of bacterial cells. This section will introduce the function of several fluorescent antibiotics used to study various aspects of bacteria, such as their regulation, behavior, binding ability, drug uptake, and mechanisms (**Figure** **4**).

#### 3.1.1 Visualizing the Cell Wall Formation

**Figure 5:**
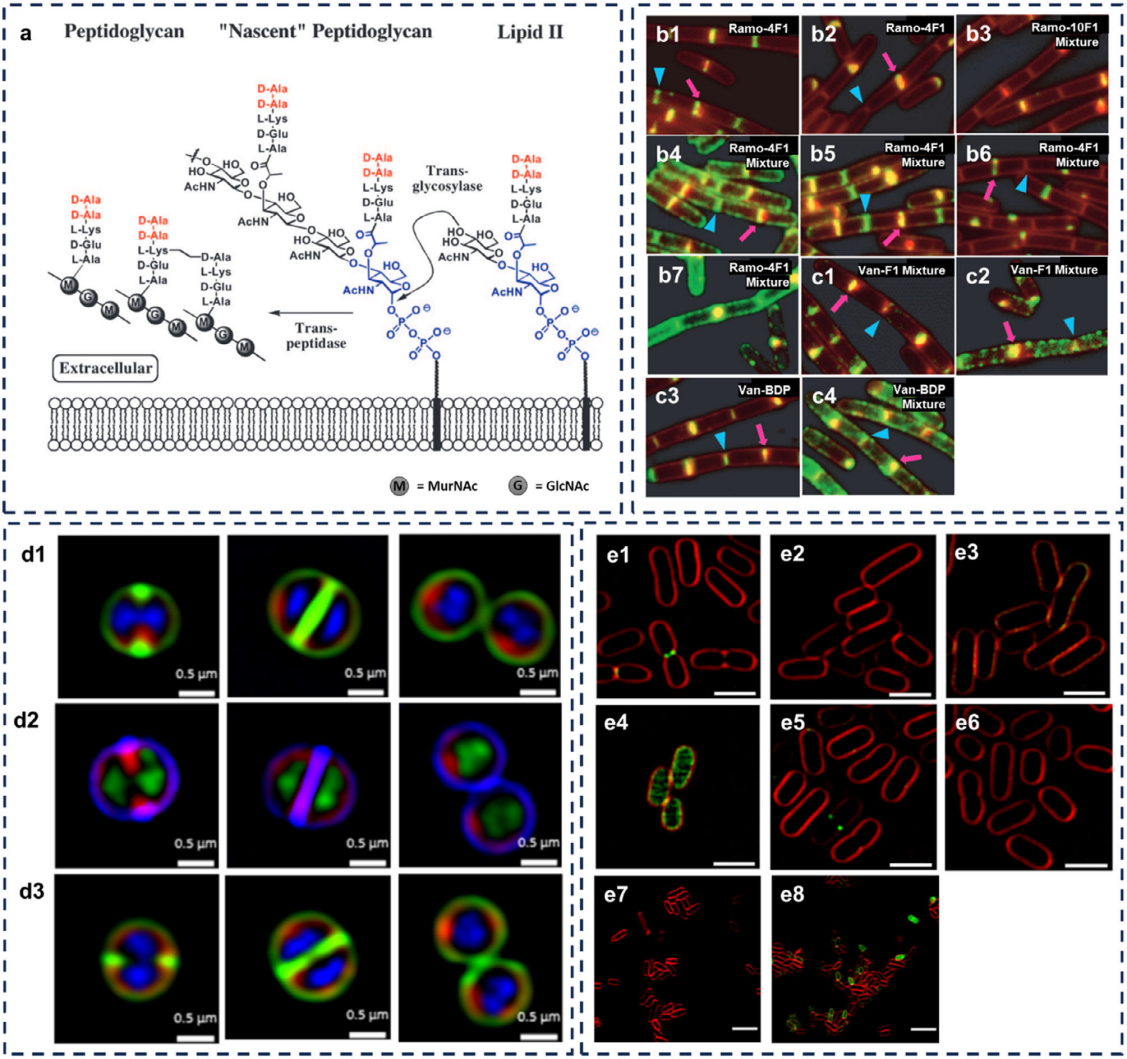
a) The extracellular stage of PG biosynthesis. Vancomycin targets D‐Ala‐D‐Ala (red); ramoplanin targets diphospho‐MurNAc (blue). In *B. subtilis*, L‐Lys is replaced with meso‐diaminopimelic acid. b) Overlays staining of *B. subtilis* PY97 with ramoplanin analogs (green) and 1‐(4‐trimethylammoniumphenyl)‐6‐phenyl‐1,3,5‐hexatriene p‐toluenesulfonate (TMA‐DPH)‐treated cells (mb, membrane stain) (red). Fluorescent images show b1) Ramo‐4F1 at 0.1 × MIC (2 μg mL^−1^) and b2) at 1.0 × MIC (20 μg mL^−1^), b3) a 1:1 mixture of Ramo: Ramo‐10F1 at 0.5 μg mL^−1^ each (higher concentrations looks similar), b4) a 1:1 mixture of Ramo: Ramo‐4F1 at 0.5 μg mL^−1^ each, b5) at 1.0 μg mL^−1^ each, and b6) at 2.5 μg mL^−1^ each; b7) Van–BDP at 0.1 × MIC (1.0 μg mL^−1^). c) Overlays staining of *B. subtilis* PY97 with vancomycin analogs (green) and TMA‐DPH‐treated cells (mb, membrane stain) (red). Fluorescent images of c1) A 1:1 mixture of Van: Van‐F1 at 0.13 μg mL^−1^ each and c2) at 0.4 μg mL^−1^ each, c3) Van–BDP at 0.4 × MIC (1.0 μg mL^−1^), and c4) a 1:1 mixture of Van: Van–BDP at 0.4 μg mL^−1^ each. Arrowheads point to old division sites (poles) and arrows to new division sites (septa). Scale bars represent 2 μm. Reproduced with permission.^[^ ^139^ ^]^ Copyright 2006, National Academy of Sciences, USA. d) SR‐SIM fluorescence imaging of *S. aureus* ATCC 25 923 cell division with different probes. d1) Green: Van‐8C‐Tz‐NBD, red: FM4‐64FX (bacterial membrane), blue: Hoechst 33 342 (nucleic acid), d2) blue: Van‐8C‐Tz‐DMACA, Red: FM4‐64FX, and green: SYTO 21 (nucleic acid), d3) green: Van‐3PEG‐Tz‐NBD, red: FM4‐64FX, and blue: Hoechst 33 342 (scale bar = 0.5 μm). e) Visualization of membrane permeabilization in Gram‐negative bacteria with Van‐3PEG‐Tz‐NBD (32 μg mL^−1^, green) and FM4‐64FX (5 μg mL^−1^, red). The scale bar represents 2 μm. SR‐SIM fluorescence imaging in *E. coli* strains e1) ATCC 25 922, e2) K12 (MB4827), and e5) CFT073, and mutant *E. coli*: e3) DC2, e4) lpxC, and e6) waaL. Visualization of membrane permeabilization in *E. coli* strain BW25113 at e7) 15 °C and e8) 37 °C. Reproduced with permission.^[^ ^56^ ^]^ Copyright 2023, Springer Nature.

PG biosynthesis in *B. subtilis* was observed using fluorescent derivative of Van (Van–FL) and BODIPY derivative of the antibiotic vancomycin (Van–BDP) and ramoplanin (Ramo‐4F1, Ramo‐10F1, and Ramo‐4BDP).^[^^139^^]^ These derivatives bind to PG precursors, allowing visualization of new PG synthesis (**Figure** **5**a–c). Both antibiotics showed concentration‐dependent staining patterns, with ramoplanin proving more effective as it did not significantly affect bacterial cell viability. Interestingly, helicoid staining patterns were observed along the cell walls of *B. subtilis*, even without the actin‐like protein Mbl. This suggests that other proteins control the spatial localization of biosynthetic complexes for new PG synthesis, providing insights into bacterial cell wall assembly and regulation.

The use of fluorescent probes based on Van, specifically Van–NBD and Van–DMACA, enabled the detection and visualization of Gram‐positive bacteria and the permeabilization of their outer membrane.^[^ ^56^ ^]^ Van probes were shown to indicate the formation of freshly synthesized PG clearly and to spatially discriminate the PG layer from the bacterial cell membrane when used for super resolution structured illumination microscopy (SR‐SIM) (Figure 5d). Flow cytometry analysis demonstrated their ability to label Gram‐positive bacteria, such as *S. aureus*, selectively, but not Gram‐negative bacteria, like *E. coli*, based on fluorescence uptake. Furthermore, the Van–NBD probe could visualize membrane permeabilization in Gram‐negative bacteria caused by genetic mutations, temperature stress, or exposure to chemical compounds (Figure 5e). This demonstrated the usefulness of the probes in assessing damage to the gram‐negative outer membrane, which was accomplished using simple flow cytometry or plate‐based tests.

#### 3.1.2 Visualizing Cell Membrane Formation

**Figure 6:**
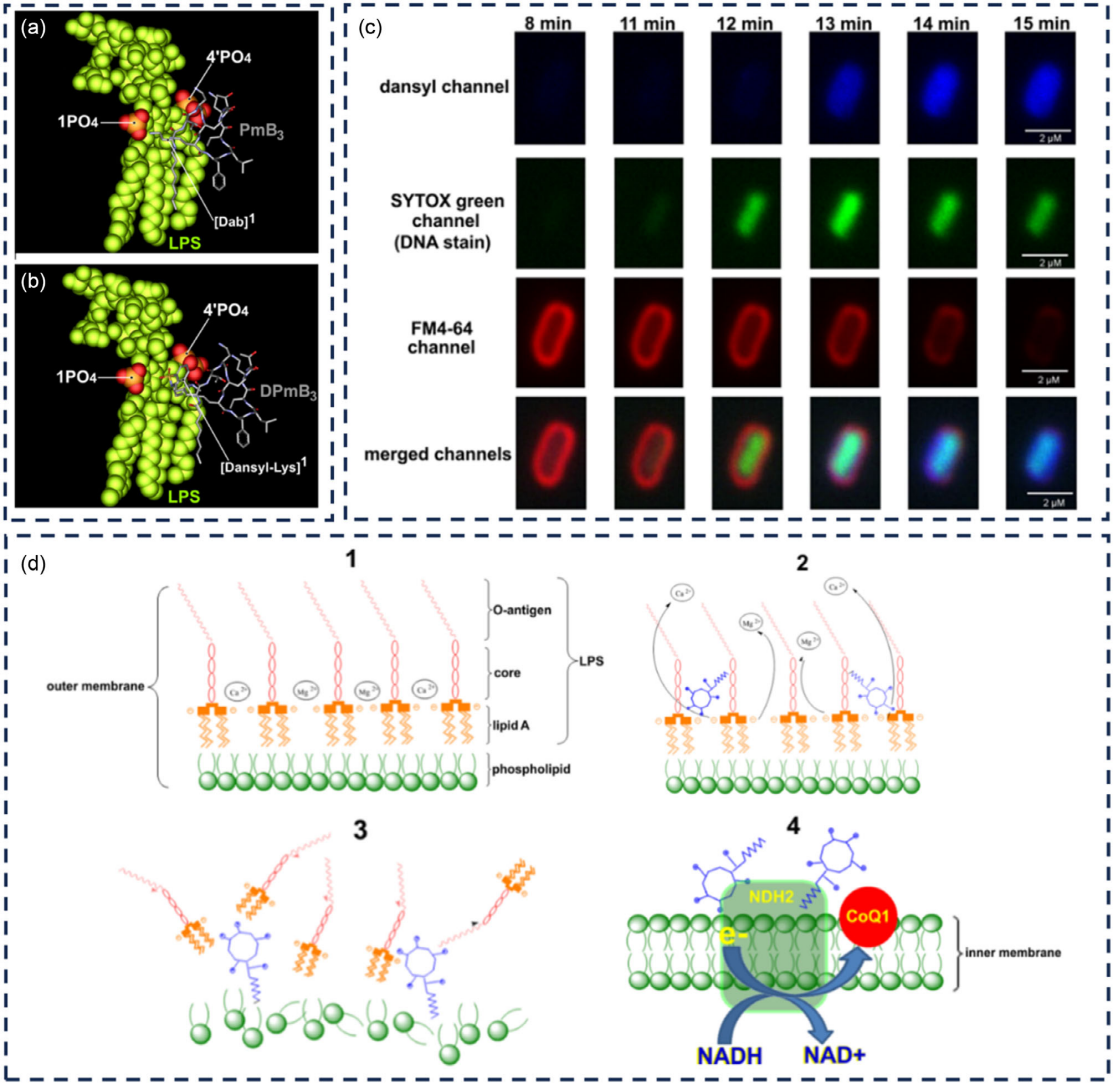
a) The molecular model of the PmB–LPS complex as determined from NMR restraints and b) the proposed structure of the DPmB_3_–LPS complex. Reproduced with permission.^[^ ^126^ ^]^ Copyright 2011, Elsevier. c) Time‐lapse imaging using laser scanning confocal microscopy showing *K. pneumoniae* ATCC 13 883 cells treated with dansyl polymyxin B at 5 × MIC, along with the membrane probe FM4−64 and the nucleic acid selective probe SYTOX green. d) A schematic diagram illustrating the mode of action of polymyxins on the outer and inner membranes of the Gram‐negative bacterial cell. Step 1: polymyxins target the outer membrane. Step 2: the positively charged polymyxins displace divalent cations that bridge adjacent LPS molecules. Step 3: the electrostatic interactions weaken the stability of the outer membrane, and the hydrophobic insertion destabilizes the outer membrane through hydrophobic expansion, damaging it. Step 4: polymyxins penetrate the inner membrane and inhibit the respiratory enzyme NDH‐2. Reproduced with permission.^[^ ^127^ ^]^ Copyright 2014, American Chemical Society.

A novel fluorescent probe, DPmB3, was developed for simple, cost‐effective quantification of LPS and polymyxin interactions,^[^ ^126^ ^]^ which is critical for understanding the initial binding of polymyxins to the bacterial outer membrane. The probe's affinity for LPS was found to be comparable to PMB and colistin, with a considerably greater binding affinity (Kd < 1 μm) than for whole cells (Kd ≈ 6–12 μm). Isothermal titration calorimetric studies demonstrated that PMB and DPmB3 binding to LPS is exothermic and enthalpically driven, primarily due to electrostatic interactions. The hydrophobic dansyl moiety of DPmB_3_ contributed a greater entropic benefit to the DPmB_3_–LPS reaction. Molecular modeling indicated a loss of electrostatic contact within the DPmB_3_–LPS complex due to steric hindrance from the dansyl[Lys]^1^ fluorophore (**Figure** **6**a,b), correlating with diminished antibacterial activity (MIC ≥ 16 μg mL^−1^). In another study, the authors used the modified dansyl–polymyxin probe for confocal laser scanning microscopy (CLSM) imaging to gain mechanistic insights into the penetration of polymyxins into Gram‐negative bacterial cells.^[^ ^127^ ^]^ The imaging data confirmed that the probe initially accumulates in the outer membrane, penetrates the inner membrane, and finally reaches the cytoplasm (Figure 6c,d). The same researchers then designed and synthesized a series of novel fluorescent PMB probes—MIPS‐9541, MIPS‐9542, MIPS‐9543, and MIPS‐9544—to investigate the localization of polymyxins within rat renal tubular cells (NRK52E cells) and to gain insights into the mechanism of polymyxin‐induced nephrotoxicity.^[^ ^128^ ^]^ The study revealed that the degree of cellular uptake of these probes varied, with MIPS‐9541 showing the highest degree of cellular uptake and the highest apoptotic index, suggesting a strong correlation between the accumulation of polymyxins in renal tubular cells and the induction of nephrotoxicity. MIPS‐9542 displayed low cellular uptake and did not induce apoptosis, even at high concentrations (1000 μm). MIPS‐9543 and MIPS‐9544, which incorporate the dansyl fluorophore at positions 7 and 6 of the polymyxin core, not only retained antimicrobial activity but also provided a balance between cellular uptake and apoptotic effects, making them valuable tools for studying the intracellular behavior of polymyxins.

#### 3.1.3 Bacterial Intracellular Behavior Studies

A fluorescence polarization (FP) assay using BODIPY‐erythromycin was developed to quantitatively measure the binding of this fluorescently labeled erythromycin to 70S ribosomes from *E. coli* and to assess the displacement of erythromycin from these ribosomes by other compounds.^[^ ^119^ ^]^ To accurately determine the dissociation constant (KD) of the BODIPY–erythromycin–ribosome complex, the authors performed a competitive binding experiment using radiolabeled erythromycin. Mixing a range of BODIPY–erythromycin concentrations with a fixed concentration of erythromycin and ribosomes and allowing the binding to occur could separate bound and free ligands through filtration. The binding data were then fitted to a cubic equation designed to solve for KD in a single binding site scenario with two competitive ligands, resulting in an estimate of KD = 20.3 ± 1.0 nm. This value represents the actual potency of BODIPY‐labeled erythromycin for ribosome binding.

A synthetic pathway of BODIPY–erythromycin probes was introduced by attaching a BODIPY fluorophore to the 4″ and 9 positions of the erythromycin structure.^[^ ^120^ ^]^ These probes demonstrated excellent binding affinity to bacterial ribosomes and were able to compete with erythromycin and other drugs that bind in the vicinity of the 50S subunit. The synthetic fluorescent probe 5 was explicitly adapted for ultrahigh‐throughput screening to identify novel ribosome inhibitors.

A series of fluorescent derivatives of macrolide antibiotic tylosin was designed by incorporating fluorophores such as rhodamine, fluorescein, Alexa Fluor 488, BODIPY FL, and NBD.^[^ ^140^ ^]^ Using FP, the authors measured a complex formation between these derivatives and ribosomes and screened the affinity of new antibiotics derivatives at the macrolide binding site. The FP method relies on the principle that binding a small, fluorescently labeled molecule to a larger molecule, such as a ribosome, will slow the rotation of the labeled molecule, increasing FP. This change can be quantitatively measured and is indicative of the binding interaction. These constants were found to be in the nanomolar range, indicating strong binding affinities and suggesting that these fluorescent derivatives can effectively mimic the binding behavior of macrolides to the ribosomal exit tunnel. The study also showed that derivatives with BODIPY FL and NBD could screen the binding of novel antibiotics to bacterial ribosomes in the macrolide‐binding site.

**Figure 7:**
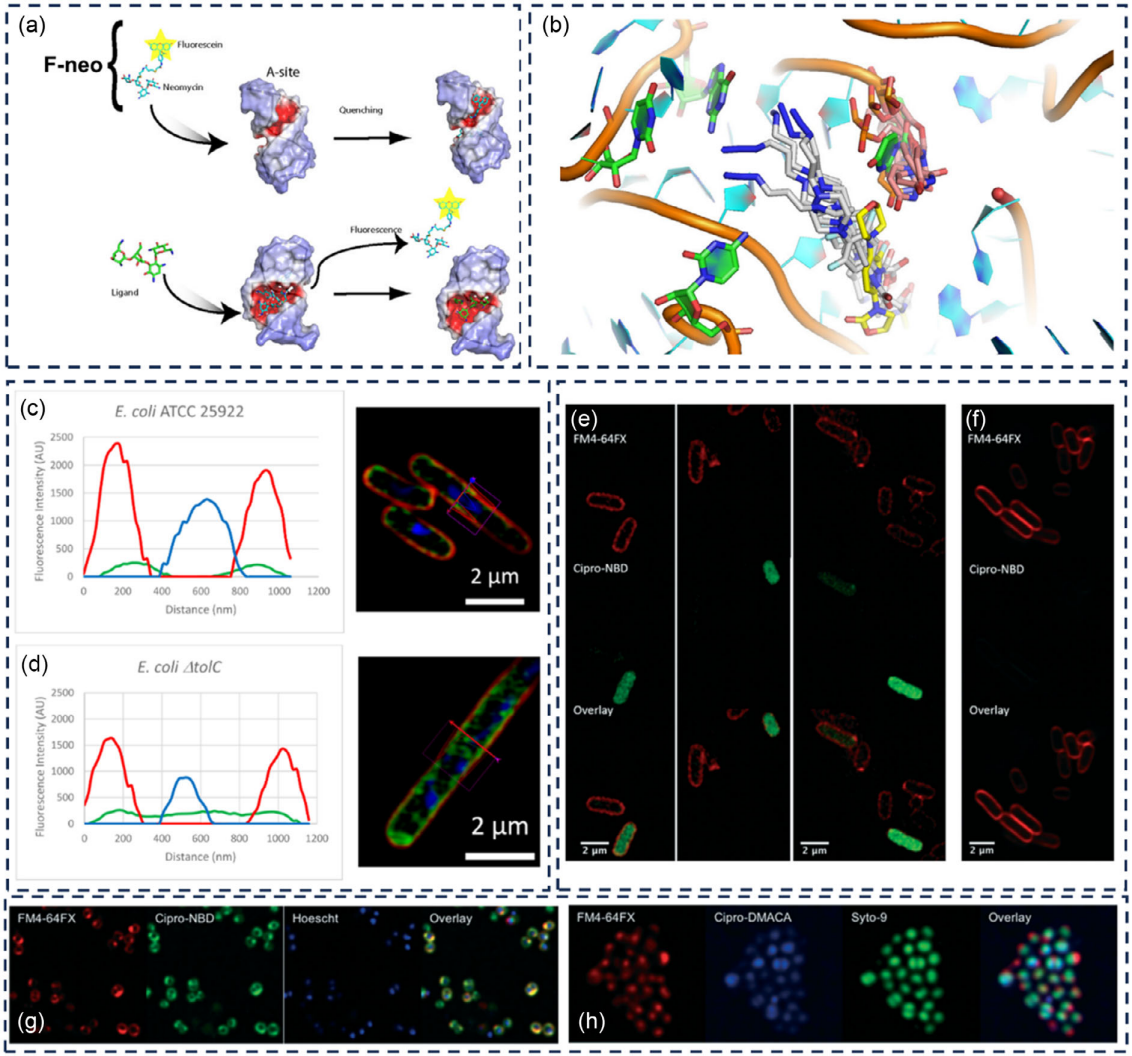
a) Schematic diagram of a competitive binding assay showing increased fluorescence as a competitive binding ligand displaces the probe. Reproduced with permission.^[^ ^121^ ^]^ Copyright 2013, Elsevier. b) In silico docking studies of the azide‐functionalized oxazolidinone within the linezolid binding site in ribosomal subunit 50S of *D. radiodurans*. Crystal structure 3DLL is shown, with key nucleotides in green and linezolid in yellow. The docked structure of the azide‐functionalized oxazolidinone is shown in grey, with conformational variations of nucleotide U2585 in orange. Reproduced with permission.^[^ ^87^ ^]^ Copyright 2014, Elsevier. c) Fluorescent imaging cross section of *E. coli* (ATCC 25 922) and d) ΔtolC *E. coli*: green, TMP‐fluorophore 4C‐Tz‐NBD; red, FM4‐64FX (bacterial membrane stain); and blue, Hoechst 33 342 (nucleic acid stain). Reproduced with permission.^[^ ^88^ ^]^ Copyright 2016, American Chemical Society. Confocal fluorescent microscopy of live *E. coli* AG102 labeled with red FM4‐64FX membrane dye and green cipro‐NBD, showing internalization: e) several fields of view and no internalization: f) without efflux pump inhibitor; live *S. aureus* labeled with g) red FM4‐64FX membrane dye, green cipro‐NBD, blue nucleic acid dye Hoechst 33 342, and overlay; h) red FM4‐64FX membrane dye, blue cipro‐DMACA, green nucleic acid dye Syto‐9, and overlay. Reproduced with permission.^[^ ^89^ ^]^ Copyright 2019, Royal Society of Chemistry.

A fluorescence‐based screening method was employed to identify antibiotics that bind to the ribosomal A‐site. The method uses a fluorescein‐conjugated neomycin molecule (F‐neo) as a probe.^[^ ^121^ ^]^ The authors used a displacement assay based on the quenching of F‐neo fluorescence upon binding to the A‐site RNA. In this assay, F‐neo displacement by other A‐site binding molecules increases fluorescence, which can be quantitatively measured using a fluorescence plate (**Figure** **7**a). This method allows for the rapid determination of the relative binding affinity of compounds to the ribosomal A‐site. Initial studies showed that F‐neo fluorescence quenching effectively measures binding to the A‐site RNA, with fluorescence changes reflecting the probe's pKa shift due to the altered electrostatic environment when moving from solvent to the A‐site negatively charged major groove.

An azide‐functionalized analog of the oxazolidinone antibiotic linezolid was synthesized through “click” chemistry. These versatile intermediates maintain their antimicrobial activity. They can be further modified to investigate antimicrobial SARs or linked to fluorophores to create fluorescent probes, such as linezolid–DMACA and linezolid–NBD.^[^ ^87^ ^]^ These probes enable real‐time visualization of bacteria and their locations within samples. Structural modeling confirmed that these probes could bind to the same ribosomal site as linezolid (Figure 7b) and were effectively used to image Gram‐positive bacteria via confocal microscopy. This approach not only aids in directly observing how antibiotics target bacteria but also in developing novel antibiotic variants. The research team subsequently created fluorescent versions of TMP to study the role of efflux pumps in drug accumulation in *E. coli* cells.^[^ ^88^ ^]^ These TMP probes were designed to inhibit DHFR without killing or inhibiting the growth of wild‐type *E. coli*. However, they showed bactericidal activity against an *E. coli* mutant strain lacking the efflux pump system outer membrane protein (tolC) component. A fluorescence assay was developed to measure the cellular accumulation of the TMP probe and assess efflux pump activity (Figure 7c,d). The assay revealed that there was reduced TMP probe accumulation in wild‐type *E. coli*, which possesses functional efflux pumps due to these pumps actively exporting the probes out of the cells. In contrast, in the efflux pump deficient *E. coli* mutant (ΔtolC), the TMP probes accumulated significantly, as evidenced by increased fluorescence signals. This difference in probe accumulation between wild‐type and mutant strains highlights the role of efflux pumps in reducing the intracellular concentration of antimicrobial agents, contributing to decreased susceptibility or resistance to these compounds. The team also used fluorescent ciprofloxacin, cipro–NBD, and cipro‐DMACA to explore bacterial penetration and efflux mechanisms.^[^ ^89^ ^]^ The researchers determined the MICs for these compounds against wild‐type strains and mutants with compromised efflux systems or outer membranes to assess the role of efflux pumps and membrane permeability in the activity of the modified antibiotics. The results showed a general increase in MIC values compared to ciprofloxacin and enhanced activity was observed against *E. coli* mutants, suggesting a decrease in antimicrobial activity due to reduced influx into the bacterial cell or increased efflux. There was also substantial improvement in the activity of a *P. aeruginosa* multiple efflux pump knockout compared to an ATTC 27 853 strain, highlighting the important role of efflux pumps in controlling the intracellular level of fluoroquinolones.

### 3.2 Selective Recognition of Gram‐Positive and Gram‐Negative Bacteria

The ability to quickly distinguish between Gram‐positive and Gram‐negative bacteria can improve the accuracy of diagnosis, the effectiveness of treatment, and the efficiency of infection control measures. Furthermore, the precise and rapid identification of bacteria allows for administering suitable antibiotics, thereby minimizing the likelihood of antibiotic resistance and improving patient prognosis. In recent years, there has been a growing trend in the development of fluorescent antibiotics, often employing Van and polymyxins, for the selective detection of Gram‐positive and Gram‐negative bacteria (Table 3). This section will discuss the practical applications and potential impact of such probes, primarily focusing on their use in clinical settings.

**Figure 8:**
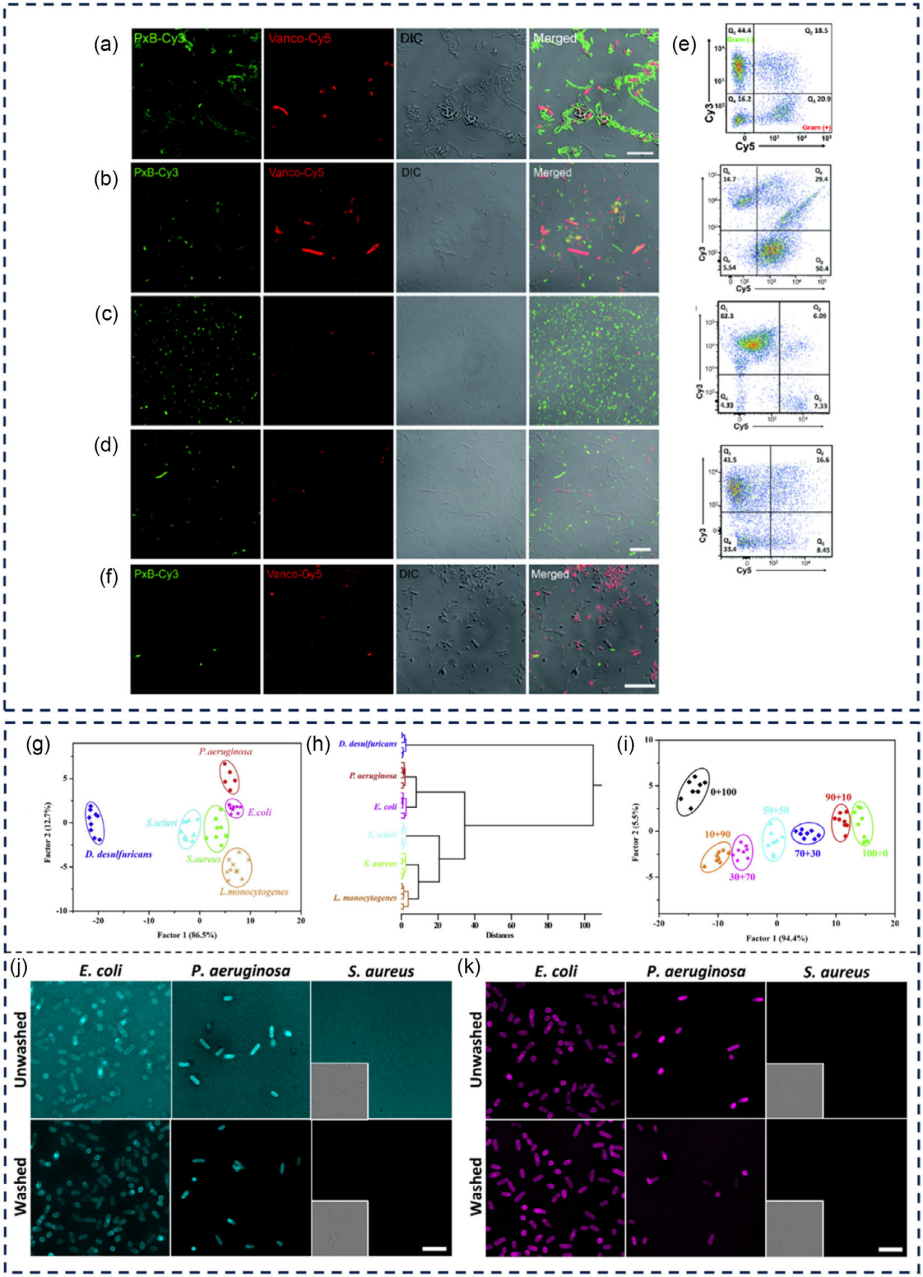
a–d) Two‐color fluorescence imaging of microbiotas from the mouse gut, human oral, soil, and crude oil, labeled with 5 μm PxB‐Cy3 for 30 min followed by 5 μm Vanco‐Cy5 for another 30 min. e) Flow cytometry analyses of the labeled microbiotas from (a–d). f) Two‐color fluorescence imaging of a heat‐fixed sputum‐smear sample labeled with PxB‐Cy3 and Vanco‐Cy5; scale bars represent 10 μm. Reproduced with permission.^[^ ^118^ ^]^ Copyright 2018, Springer Nature. g) Canonical score plot for bacterial discrimination using the CDs‐based sensor array showing clear separation of six bacterial types. h) Hierarchical cluster analysis dendrogram of six different bacteria. i) Canonical score plot for the CDs‐based fluorescent sensor array against the bacteria for *D. desulfuricans* and *L. monocytogenes* mixtures at varying ratios (100 + 0, 90 + 10, 70 + 30, 50 + 50, 30 + 70, 10 + 900, and 0 + 100). Reproduced with permission.^[^ ^141^ ^]^ Copyright 2019, Elsevier. Confocal laser scanning microscopy images of live bacteria treated with j) meroBA‐PMX (5 μm) and k) meroSO‐PMX (5 μm) under “Unwashed” and “Washed” conditions. Insets for *S. aureus* show brightfield images. The scale bar represents 5 μm. Reproduced with permission.^[^ ^136^ ^]^ Copyright 2021, Elsevier.

A cell‐selective labeling method was developed to distinguish Gram‐negative and Gram‐positive bacteria in the mouse gut by exploiting their unique cell wall components.^[^ ^117^ ^]^ This approach combines metabolic labeling of Gram‐negative bacterial LPS with a clickable azidosugar and direct labeling of Gram‐positive bacteria with a Van‐derivatized fluorescent probe. The method was demonstrated through two‐color fluorescence imaging, showing distinct labeling of Gram‐negative and Gram‐positive gut microbiotas in mice. This strategy enables selective imaging of bacterial communities, facilitating research in gut microbiota and potentially other bacterial studies without genetic manipulation or the cultivation of specific bacterial species. Subsequently, the same researchers devised a fluorescent probe based on PMB for selective labeling of Gram‐negative bacteria within live microbiota samples.^[^ ^118^ ^]^ They synthesized a PMB‐Cy3 conjugate and confirmed its specificity for labeling Gram‐negative bacteria. Combined with a previously developed Gram‐positive‐specific fluorescent probe, they achieved two‐color imaging of Gram‐positive and Gram‐negative bacteria in various living microbiotas, with high selectivity and coverage (**Figure** **8**a–f). This method provides a versatile tool for distinguishing Gram‐positive and Gram‐negative bacteria in basic research and clinical settings.

A fluorescent UCNPs derivative, NaYF4:Yb, Tm, Fe–PMP (PMP–UCNPs), was developed to detect *E. coli*.^[^ ^111^ ^]^ The detection mechanism relies on the specific binding of PMB to LPS, facilitated by the self‐promoting uptake mechanism. Upon incubation with *E. coli*, PMB–UCNPs formed complexes with the bacteria, which were then separated and measured for fluorescence intensity at 801 nm under 980 nm light excitation. A linear correlation was observed between the fluorescence intensity and *E. coli* concentration, with a limit of detection (LOD) of 36 colony‐forming units (CFU). The specificity of the PMB–UCNP probe was evaluated by detecting *E. coli* in the presence of Gram‐positive bacteria, *S. aureus*, and *B. subtilis*, revealing some positive deviation due to nonspecific adsorption. The practical application was demonstrated using soybean milk spiked with varying concentrations of *E. coli*, achieving a linear relationship between fluorescence intensity and the different concentrations of *E coli*. The results from the proposed method were consistent with plate‐counting analyses, with a relative standard deviation of ≤5.22%. The entire detection process took less than 2.5 h, highlighting the efficiency and applicability of the PMB–UCNPs fluorescent probe for rapid and sensitive detection of *E. coli* in complex matrices.

An array of antibiotic‐functionalized semiconducting Pdots was used to identify various bacterial strains.^[^ ^114^ ^]^ These Pdots exhibit a fluorescence intensity that correlates with the concentration of bacteria. Van–Pdots are designed to attach to Gram‐positive bacteria such as *S. aureus*, and PMB‐Pdots (PB–Pdots) are aimed at Gram‐negative bacteria like *P. aeruginosa*. The increase in fluorescence indicates the formation of a complex between the bioprobe and bacterial cells. Van–Pdots could detect as few as 3.1 × 10^4^ CFU mL^−1^ of *S. aureus*, while PB–Pdots were sensitive to 1.0 × 10^4^ CFU mL^−1^ of *P. aeruginosa*. The study also discovered that Van–Pdots and PB–Pdots could selectively kill Van‐sensitive *S. aureus* and PMB‐sensitive *P. aeruginosa* under white light within 1 h. At low concentrations, bioconjugated Pdots showed good biocompatibility. Hence, these probes presented an alternative to traditional methods like chlorination and UV sterilization and could markedly improve bacterial detection and treatment, even for antibiotic‐resistant bacteria. In another study by the same researchers, a fluorescence sensor array was developed using CDs functionalized with three different receptors: boronic acid (BA‐CDs), polymyxin (PM‐CDs), and Van (Van–CDs).^[^ ^141^ ^]^ These CDs were designed to bind to bacteria with varying affinities, creating a unique molecular fingerprint for each type of bacteria based on the fluorescence response pattern generated by the CDs. The sensor array effectively discriminates six different bacteria studied using linear discrimination analysis of the response patterns (Figure 8g). Hierarchical cluster analysis was used to analyze the similarities among various bacterial species, resulting in six distinct clusters representing different bacterial types. Gram‐negative bacteria *P. aeruginosa* and *E. coli* were closely matched, as were Gram‐positive bacteria *L. monocytogenes* and *S. aureus*, with *Staphylococcus sciuri* also grouped with the latter (Figure 8h). The bacteria mixtures of *Desulfovibrio desulfuricans* and *Listeria monocytogens* with different ratios were also well separated without any overlap (Figure 8i). These arrays can also effectively identify bacteria in real samples, such as tap water.

To continue the work of Merocy–Van (as discussed previously), the same class of merocyanine fluorophores was used to prepare redshifted polymyxin (PMX)‐based imaging probes, which were analyzed for their ability to fluorescently and selectively label Gram‐negative bacteria.^[^^136^^]^ The study investigated two probes, meroBA‐PMX and meroSO‐PMX, for their ability to image bacteria using a rapid and straightforward labeling protocol. These probes’ excitation and emission spectrum showed that meroSO‐PMX had optimal excitation at 590 nm and emission at 630 nm, distinguishing it from the spectra of NBD and tissue autofluorescence. The environmentally sensitive fluorescence of these compounds, which intensifies upon binding to the lipophilic bacterial membrane, was confirmed by the increase in fluorescence observed with the addition of DMSO in saline. MeroSO‐PMX exhibited a more than 20‐fold increase in fluorescence enhancement, aligning with measurements performed on nonconjugated dyes. Confocal imaging revealed that both meroBA‐PMX and meroSO‐PMX could proficiently label Gram‐negative bacteria (*E. coli* and *P. aeruginosa*) but not Gram‐positive bacteria (*S. aureus*) without requiring a washing step to eliminate nonspecific background fluorescence, particularly for meroSO‐PMX. However, imaging of meroBA‐PMX benefited from gentle washing to enhance signal clarity by removing the unbound probes (Figure 8j,k). Nevertheless, this study highlights the potential of these probes for rapid, wash‐free bacterial imaging with excellent Gram selectivity.

A two‐step labeling process for detecting a panel of Gram‐positive bacteria, including species such as *S. aureus*, *S. pneumoniae*, *S. epidermidis*, and *E. faecalis*, was established by Chung and coworkers.^[^ ^99^ ^]^ These bacteria were incubated with vancomycin conjugated TCO (vanc‐TCO) or daptomycin conjugated TCO (dapt‐TCO) and then reacted with MFNP–Tz through a rapid cycloaddition reaction. The study identified 20 μm as the optimal dose of vanc‐TCO for bacterial labeling, with higher concentrations leading to reduced efficacy due to the bactericidal effects of vanc‐TCO. The fluorescent intensities increased proportionally with vanc‐TCO concentration for Gram‐positive bacteria, while Gram‐negative bacteria (*E. coli*, *P. aeruginosa*, and *K. pneumoniae*) showed negligible signals. This two‐step method exhibited up to sixfold higher cellular reactivity than the direct conjugation approach (MFNP‐vanc). The LOD was as low as 1300 CFU for *S. aureus* and 35 100 CFU for *S. epidermidis*. These assays were completed within 30 min, with multimodal detection reliability validated by fluorescence and micro nuclear magnetic resonance (μNMR) correlation. While dapt‐TCO was effective in bacterial labeling, its efficacy was slightly lower than vanc‐TCO, likely due to steric hindrance and partial penetration into bacterial membranes. Despite this, it showed substantial increases in fluorescent intensities (up to 220%) and cellular relaxivity reached 1.32 s^−1^ [CFU μL^−1^]^−1^. This bioorthogonal method was also successfully applied to intracellularly detect bacteria within live macrophages. Control experiments confirmed that the results were not due to normal phagocytosis, as neither *E. coli* nor bacteria‐free macrophages showed intracellular MFNPs. This method could be adapted for other antibiotics and bacterial targets, offering a new approach to the selective detection of bacteria.

### 3.3 Detection and Eradication of Antibiotic‐Resistant Bacteria and Biofilms

The rise of antibiotic‐resistant bacteria is a significant global health concern. These bacteria have evolved and developed defense mechanisms to evade the effects of antibiotics and render traditional treatments ineffective.^[^ ^142^ ^]^ Compounding this issue are bacterial biofilms—complex communities of bacteria encased in a protective matrix of biomolecules known as extracellular polymeric substances, often attached to surfaces. Biofilms exhibit a high degree of antibiotic tolerance, mainly because most antibiotics cannot reach the deepest layers of the biofilms and also due to genetic alterations within the biofilm.^[^ ^143^ ^]^ Consequently, developing new molecules, compounds, and materials capable of penetrating and eliminating biofilms created by MDR bacteria inevitably becomes a formidable challenge. This section will present the latest fluorescence‐based antibiotics designed to detect and eradicate MDR bacteria and biofilms.

**Figure 9:**
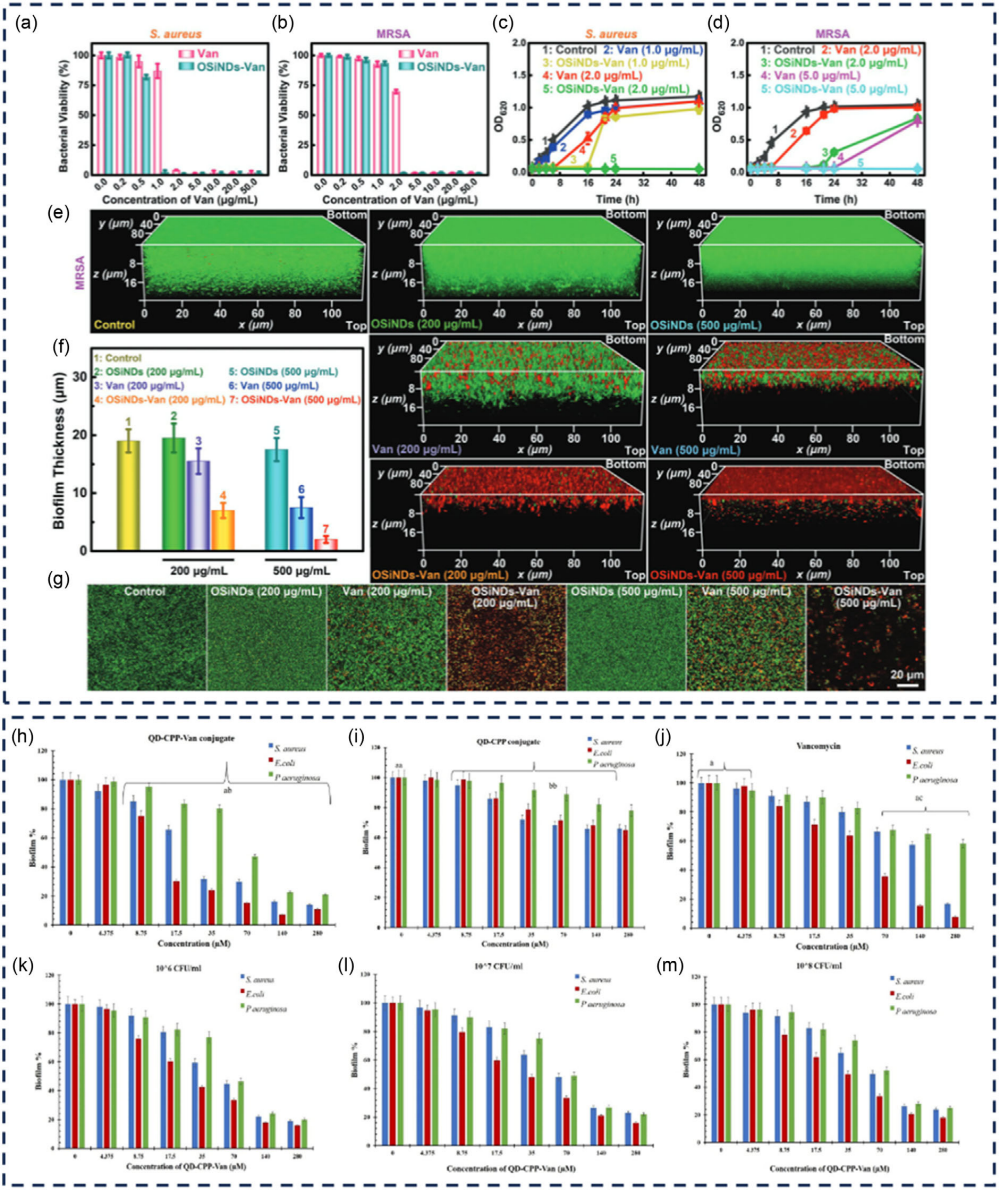
Viabilities of planktonic a) *S. aureus* and b) MRSA cells after 12 h of treatment with different concentrations of free Van or OSiNDs‐Van. OD_620_ values of c) *S. aureus* and d) MRSA suspensions treated with different concentrations of free Van or OSiNDs‐Van for different durations, compared to controls. e) 3D‐reconstructed confocal fluorescence images of MRSA biofilms treated with OSiND–, Van–, and OSiND–Van with an untreated biofilm as the control. Before imaging, a LIVE/DEAD BacLight bacterial viability kit stained the biofilms to visualize the live (green) and dead (red) bacteria. f) Statistical analysis of biofilm thicknesses in (e). g) Confocal fluorescence images of the MRSA cells at the bottoms of biofilms after various treatments stained with a LIVE/DEAD *Bac*Light bacterial viability kit. Reproduced with permission.^[^ ^138^ ^]^ Copyright 2019, John Wiley and Sons. Biofilm inhibitory graphs of *S. aureus*, *E. coli*, and *P. aeruginosa* in the presence of h) QD‐CPP‐Van, i) QD‐CPP conjugate, and j) Van only. k) Effect of QD‐CPP‐Van conjugate on the bacterial density of 10^6^ CFU mL^−1^, l) 10^7^ CFU mL^−1^, and m) 10^8^ CFU mL^−1^. Data plotted as mean ± SD and *n* = 3, and lowercase letters indicate significant differences among bacterial strains for biofilm inhibition using nanoformulation (*p* < 0.05). Similar letters indicate no significant difference (*p* > 0.05). Reproduced with permission.^[^ ^144^ ^]^ Copyright 2023, John Wiley and Sons.

A multifunctional nanoplatform, Van‐modified nanodots (OSiNDs–Van), was constructed for imaging and eliminating MDR bacteria and their biofilms.^[^ ^138^ ^]^ The study compared the antibacterial activities of free Van and OSiNDs–Van against Gram‐positive bacteria, specifically, *S. aureus* and drug‐resistant MRSA. Both agents reduced the viability of these bacteria in a dose‐dependent manner, with OSiNDs–Van showing superior efficacy, completely inactivating *S. aureus* and MRSA at lower concentrations than free Van (**Figure** **9**a,b). The MICs of OSiNDs–Van were lower than those of Van for both bacteria, indicating enhanced antibacterial potency. Long‐term antibacterial efficacy was also assessed, revealing that OSiNDs–Van could inhibit bacterial growth for up to 48 h (Figure 9c,d). Furthermore, the study explored the anti‐biofilm activity of OSiNDs–Van against *S. aureus* and MRSA biofilms. Confocal fluorescence microscopy imaging showed that OSiNDs–Van dramatically destroyed both types of biofilms, reducing their thickness and effectively killing bacteria within the biofilms as opposed to the limited efficacy of free Van (Figure 9e–g). This enhanced performance was attributed to the deep biofilm penetration capability of the nanocarriers combined with the antibacterial activity of Van. In vivo tests using an MRSA‐infected mouse model confirmed the superior antibacterial ability of OSiNDs–Van compared to free Van, showcasing its potential as a more effective treatment option for bacterial infections, including those resistant to conventional antibiotics.

Another study evaluated the antibiofilm efficacy of Van and its conjugate with QDs and cell‐penetrating peptides (QD‐CPP‐Van) against both Gram‐positive and Gram‐negative microorganisms.^[^ ^144^ ^]^ The QD‐CPP‐Van conjugate effectively eradicated biofilms formed by Gram‐negative pathogens such as *E. coli* and *P. aeruginosa*, as well as Gram‐positive *S. aureus* (Figure 9h–m). Utilizing a sublethal concentration (1 X MIC) for 24 h resulted in no observable bacterial colonies, indicating the potent biofilm inhibitory property of the conjugate across a bacterial density range of 10^5^–10^8^ CFU mL^−1^. Specifically, the QD‐CPP‐Van combination inhibited nearly 80% of biofilm formation at varying concentrations depending on the bacterial strain, with significant biofilm inhibition even at high bacterial densities. Interestingly, while the CPP alone did not inhibit biofilm growth, the QD‐CPP‐Van combination was notably effective. However, concerns remain regarding the potential leaching of Cd^2+^ ions from the QDs in long‐term applications. Further studies of the effects of QD‐CPP and QD‐CPP‐drug conjugate on various mammalian cell lines indicated no cytotoxic activity against tested cell lines. However, increased CPP concentration on the QD surface was associated with increased toxicity due to higher cellular uptake. Nevertheless, QD‐CPP‐Van conjugates can be considered a powerful tool for inhibiting bacterial biofilm growth, offering a promising approach to addressing bacterial infections.

### 3.4 Fluorescent Antibiotics as Photodynamic Therapy and Treatment of Infections

The distinct capacity of AIE to transition from a non‐emissive or faintly emissive state when dispersed to a highly fluorescent state upon aggregation has been leveraged to advance photodynamic therapy (PDT) and exhibited potential for combating bacterial infections.^[^ ^145^, ^146^, ^147^, ^148^ ^]^ PDT works by activating photosensitizers with light, triggering the production of ROS. These ROS damage cellular structures, potentially leading to cell death. AIE‐active photosensitizers are particularly useful for antimicrobial PDT because their fluorescence and ROS generation are enhanced in the aggregated state. For bacterial treatment, AIE luminogens (AIEgens) are designed to selectively target bacteria, often by introducing positive charges or specific ligands with a high affinity for bacteria. These AIEgens can be activated by light to selectively eradicate pathogenic bacteria, including antibiotic‐resistant strains, without harming mammalian cells.^[^ ^149^, ^150^, ^151^, ^152^ ^]^ For example, a novel light‐up probe with AIE characteristics, named AIE‐2Van, was developed for the selective recognition, naked‐eye detection, and image‐guided photodynamic killing of Gram‐positive bacteria, including VRE strains.^[^ ^91^ ^]^ AIE‐2Van exhibits weak fluorescence in aqueous solutions but shows enhanced emission upon binding to Gram‐positive bacteria such as *B. subtilis*. The unique AIE characteristics of AIE‐2Van enable the direct naked‐eye visualization of Gram‐positive bacteria in solutions when used at high concentrations. Graphene oxide helped pre‐quench the background fluorescence of AIE‐2Van under UV light, allowing for the differentiation of Gram‐positive bacteria from Gram‐negative bacteria and VRE strains based on the emission color. AIE‐2Van exhibited high toxicity to *B. subtilis* in the absence of light, effectively eliminating almost all *B. subtilis* with a 10 mm concentration of the probe. Even at a concentration as low as 2 mm, the probe could eradicate all *B. subtilis* bacteria under white light. However, AIE‐2Van shows almost zero toxicity toward *E. coli* in the dark or upon light irradiation. It also showed low killing efficiencies toward VRE strains in the dark due to their resistance to Van. Furthermore, with light irradiation, the photodynamic treatment enhanced the killing effects, with bacteria lethality of over 70 and 90% for *E. faecium* (Van A, genotype, ATCC 51 559) and *E. faecalis* (Van B, ATCC 51 299), respectively, when a 10 mm probe is used. Another AIE‐based bioprobe (AIE‐DCM‐2polymyxinB) by conjugation of a far‐red/near‐infrared emissive AIEgens and two PMB peptides was aimed to selectively visualize Gram‐negative bacteria and effectively kill them via photodynamic treatment.^[^ ^93^ ^]^ The specificity of the bioprobe was demonstrated by its selective staining and red fluorescence emission under UV light in Gram‐negative *E. coli* and *S. enteritidis*. In contrast, Gram‐*positive E. faecalis* and *S. aureus* mutants exhibited no fluorescence. Moreover, the bioprobe demonstrated inherent toxicity against Gram‐negative bacteria without light, possibly attributable to the PMB element. Under white light, AIE‐DCM‐2polymyxinB achieved over 95% lethality against *E. coli* and *S. enteritidis*, highlighting its potential as a PDT agent for targeting Gram‐negative bacteria. This work provides a promising avenue for developing highly efficient, targeted antibacterial strategies.

A simple and specific divalent Van–porphyrin was developed utilizing Van as the targeting ligand and porphyrin as the photosensitizer.^[^ ^122^ ^]^ The study explored the effectiveness of Van–porphyrin derivatives, specifically monovalent (3a) and divalent (3b) Van derivatives, in selectively recognizing and imaging Gram‐positive bacteria, particularly *B. subtilis* and VRE strains, through fluorescent imaging techniques. Incubation with these derivatives led to a notable increase in fluorescence in *B. subtilis*, with 3b showing stronger fluorescence than 3a, indicating a higher binding affinity of 3b to the bacterial surface. Nonetheless, lower concentrations did not yield noticeable fluorescence in VRE strains, with effective fluorescent imaging only achievable at higher concentrations of 3b (10 mm), indicating a reduced binding affinity of Van–porphyrin to VRE compared to Van‐sensitive strains. The study also investigated VRE's photodynamic inactivation under white light exposure by these photosensitizers. The research revealed that augmenting the concentrations of photosensitizers improved the efficacy of bacterial killing for both *E. faecium* (VanA genotype, ATCC 51 559) and *E. faecalis* (VanB genotype, ATCC 51 299) strains, with 3b exhibiting the highest antibacterial action. Notably, 3b achieved ≈95% bacterial lethality in VanB upon irradiation, demonstrating its potent antibacterial effect. The study concluded that the divalent Van–porphyrin, 3b, due to its efficient multivalent interactions, could be an effective photoactive antibacterial agent against both Van‐sensitive and VRE strains, highlighting its potential for PDT.

Photosensitive PMP–CON were developed for the targeted identification and synergistic PDT in a single application against resistant Gram‐negative bacteria.^[^ ^95^ ^]^ The study explored the photodynamic antimicrobial therapy (PDAT) efficacy of PMB–CON, a conjugated molecule, against *kanamycin‐resistant E. coli* (*KanaR E. coli*). It was found that PMB–CON and CON can generate ROS under light irradiation, with PMB–CON producing more ROS than CON, indicating the contribution of PMB in PMB–CON to ROS generation. The photodynamic antimicrobial activity of PMB–CON was demonstrated through a considerable reduction in the survival fraction of *KanaR E. coli* under light irradiation, showcasing better photodynamic antibacterial properties compared to CON. The study highlighted that PMB modification effectively enhances the photodynamic antibacterial activity of CON. Further investigation into the photodynamic killing effect of CON and PMB–CON at different concentrations revealed a decrease in *kanaR E. coli* viability under light irradiation. PMB–CON was 25.6 times more efficient than CON in photodynamically killing *KanaR E. coli*. This indicates the selective and high‐efficiency photodynamic killing ability of PMB–CON. Direct observation using SYTOX^TM^ green dye and CLSM imaging confirmed the capability of PMB–CON in specific binding and antibacterial photodynamic treatment. A comparison between an antibody‐conjugated CON (Ab‐CON) and PMB–CON showed that PMB–CON notably decreases the survival rate of *KanaR E. coli* under light irradiation, with a photodynamic killing rate of 98.0%, much higher than 29.4% of Ab‐CON, suggesting a synergistic photodynamic killing activity of PMB–CON against *KanaR E. coli*, attributed to both CON and PMB components. The study also demonstrated PMB–CON's selective photodynamic antibacterial activity by comparing its effects on *KanaR E. coli*, *S. aureus*, and *C. albicans*, showing excellent selectivity for *KanaR E. coli*. In vivo, tests on mice infected with *KanaR E. coli* revealed that PMB–CON associated with light treatment significantly enhanced wound healing and reduced bacterial counts, indicating its potent photodynamic antimicrobial activity in vivo.

### 3.5 Innovative Photoactivatable Antibiotics in Photopharmacology and Pharmacodynamics

Photopharmacology is a growing field that employs photoswitchable ligands to allow for light‐dependent pharmacological activity. By integrating photoswitches into the exterior of existing drugs, improved spatial and temporal selectivity can be achieved, which can subsequently be harnessed for new concepts in therapy.^[^ ^153^, ^154^ ^]^

Valema and colleagues developed a series of quinolone analogs modified with photoswitchable azobenzene moieties, allowing reversible isomerization between active (cis) and inactive (trans) states upon light exposure.^[^ ^155^ ^]^ UV light (365 nm) triggers the cis isomer transition, substantially enhancing antibacterial activity against Gram‐negative *E. coli* and Gram‐positive *Micrococcus luteus* (*M. luteus*). In contrast, visible light reverses the antibiotic to the inactive transform. Among modified quinolones, compound 2, the photoswitchable quinolone, where R is 4‐methoxy‐3‐methyl benzene, showed the most pronounced difference in activity, with an eightfold increase in efficacy for *M. luteus* upon switching. Moreover, the active cis form can thermally revert to the inactive transform over a few hours, which prevents environmental accumulation and reduces the risk of resistance development. A pharmacodynamic study with photoswitchable quinolones showed that these modified antibiotics exert their bactericidal effect during the exponential growth phase, consistent with their mechanism of binding to DNA gyrase and inhibiting replication. The method also enabled localized bacterial inhibition and spatial patterning using light, demonstrated by selectively inhibiting bacterial growth in illuminated areas on agar plates.

The same research group then introduced a single‐step modification method for converting ciprofloxacin into two photoswitchable derivatives: ciprofloxacin (spiropyran‐modified) and ofloxacin (azobenzene‐modified).^[^ ^156^ ^]^ These antibiotics exhibit different biological activities depending on their light‐induced isomeric states. The study found that spirofloxacin transitions between its spiropyran (inactive) and merocyanine (active) states exhibited higher antibacterial activity against *E. coli* upon UV‐light irradiation (365 nm). In contrast, azofloxacin undergoes *trans*‐cis isomerization, with the transform exhibiting significantly higher antibacterial against *M. luteus*. The activity of azofloxacin was nearly 50 times greater than unmodified ciprofloxacin for *M. luteus*. Bacterial patterning experiments were performed using spirofloxacin to selectively inhibit bacterial growth of *E. coli* only in illuminated areas, indicating that significant spatiotemporal resolution can be obtained with photoswitch–drug conjugates.

Shchelik et al. demonstrated the development of light‐activated derivatives of Van and cephalosporin by incorporating photocleavable and PEGylation modifications.^[^ ^157^ ^]^ The antibiotics could be activated precisely with UV light at 365 nm, effectively restoring antibacterial properties after “uncaging” the modified antibiotics to their active forms. Van derivatives displayed excellent activity against Gram‐positive bacteria strains, including *B. subtilis* and vancomycin‐susceptible and methycillin‐resistant *Staphylococcus aureus* (VSSA and MRSA). At the same time, cephalosporin showed significant efficacy against Gram‐negative bacteria, such as *E. coli* and *P. aeruginosa*. The efficient photocleavage of both derivatives reaches 70% conversion within 5–6 min of UV irradiation at 365 nm without forming undesired by‐products. The study suggests that adding PEG chains suppressed antibiotic activity, proving their effectiveness in steric blocking.

These studies demonstrated the potential of photoswitchable antibiotics as a groundbreaking strategy for targeted antibacterial therapies. By enabling localized activation post‐systemic absorption, this approach enhances antibiotic precision, reduces systemic side effects, and supports precision medicine. Furthermore, it offers a promising solution to minimize antibiotic residues in the environment, thereby mitigating concerns about bacterial resistance.

### 3.6 Real‐Time In Vivo and Ex Vivo Imaging of Bacterial Infections

Real‐time in vivo imaging is a cutting‐edge technique that allows researchers and clinicians to observe and monitor bacterial infections. This approach typically employs fluorescent probes or bioluminescent markers targeting bacterial cells. Once these markers are introduced into the sites of interest, they bind to bacteria and emit radiation that can be detected and imaged using specialized equipment. This section will explore the developments of current fluorescent antibiotics used for tracking bacterial infections in various organs.

The in vivo efficacy of Vanco‐800CW was demonstrated in a mouse model of myositis induced by bioluminescent *S. aureus*.^[^ ^115^ ^]^ Vanco‐800CW specifically colocalized with the bioluminescence from the *S. aureus* strain, effectively distinguishing *S. aureus* infections from *E. coli* infections or sterile inflammation. Biodistribution analysis revealed strong fluorescence signals in the kidneys and urine, with a minor signal in the liver, suggesting that vanco‐800CW is metabolized by the liver and excreted via the kidneys, similar to native Van and unconjugated IRDye 800CW. Importantly, infection site signals were distinguishable from those of the kidneys, bladder, and liver, with no significant signal detected in other organs, including the bowel, indicating that gut microbiota would not interfere with bacterial infection imaging.

**Figure 10:**
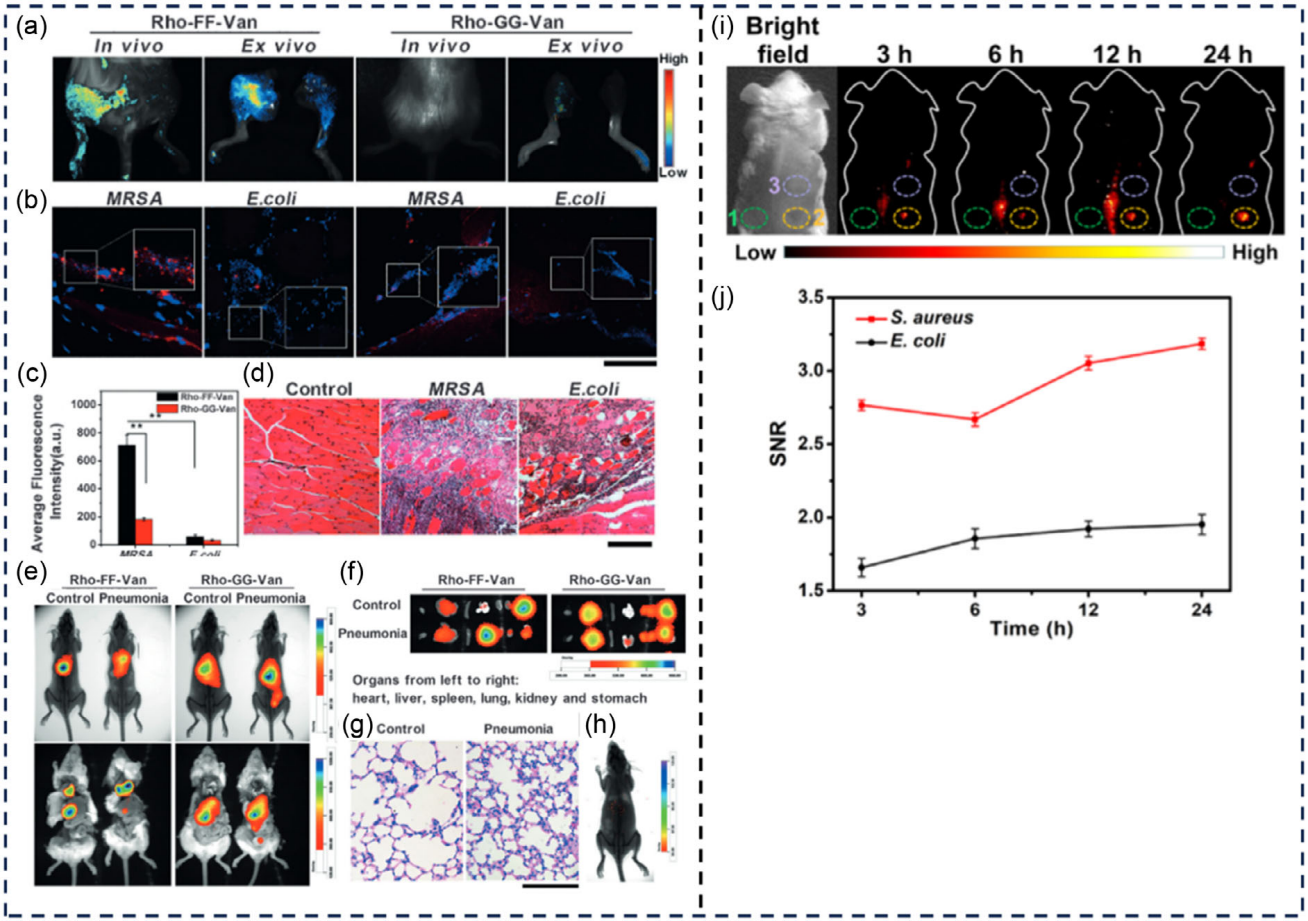
a) In vivo and ex vivo imaging of infected mouse with MRSA‐induced myositis in the left hind leg and *E. coli*–induced myositis in the right hind leg, 2 h after intravenous injection of Rho‐FF‐Van or Rho‐GG‐Van (1.25 mg kg^−1^). b) CLSM images of infected tissue slices. Blue: 4′,6‐diamidino‐2‐phenylindole (DAPI), red: rhodamine, and inset: enlarged images. c) Quantitative analysis of ex vivo fluorescence intensity. Data plotted as mean ± SD, *n* = 3, and **p < 0.01. d) Hematoxylin and eosin (H&E) images of noninfected and infected sites. Scale bars represent (c) 50 mm and (d) 200 mm. e) In vivo imaging of mice with MRSA pneumonia, showing the overlay of X‐ray and isotope signals (upper two images) and the overlay of bright field and isotope signals (lower two images) after the opening of the thoracic and abdominal cavity). f) Ex vivo images of major organs 0.5 h after intravenous administration of ^125^I‐Rho‐FF‐Van or ^125^I‐Rho‐GG‐Van (1.25 mg kg^−1^). g) H&E staining of the lung 24 h after establishing MRSA‐induced pneumonia. Scale bar represents 100 mm. h) In vivo distribution of ^125^I‐Rho‐FF‐Van in mice 24 h after intravenous injection, showing the almost complete elimination from the body. Reproduced with permission.^[^ ^158^ ^]^ Copyright 2017, John Wiley and Sons. i) NIR‐II fluorescence images of bacteria‐infected mice at different time points after intravenous injection of DCNPs‐Van/Cu_2−*x*_Se showing 1) *E. coli* infected site, 2) *S. aureus* infected site, and 3) normal tissue as a control site. These findings are significant for developing advanced clinical diagnostics. j) Calculated signal‐to‐noise ratio (SNR) for *S. aureus* and *E. coli* infected site in (i). Reproduced with permission.^[^ ^160^ ^]^ Copyright 2022, American Chemical Society.

The efficacy of a Van‐ and rhodamine‐modified peptide derivative (Rho‐FF‐Van) for in vivo detection of Gram‐positive bacterial infections was explored using a mouse model with MRSA‐induced myositis on one limb and *E. coli*‐induced infection on the other.^[^ ^158^ ^]^ Rho‐FF‐Van generated significant fluorescence at MRSA infection sites, distinguishing MRSA infections from *E. coli* infections and sterile inflammation, with a 3.9‐fold higher fluorescence intensity for MRSA than the control probe rhodamine‐glycine‐glycine‐vancomycin (Rho‐GG‐Van) (**Figure** 10a–d). This was attributed to the superior binding efficiency of Rho‐FF‐Van due to its multivalent structure enhancing cooperative binding energy with d‐Ala‐Ala motifs on bacterial surfaces. Additionally, iodine‐125 labeled Rho‐FF‐Van (^125^I‐Rho‐FF‐Van) was used to detect MRSA in pneumonia‐bearing mice (Figure 10e–h), showing that isotope signals were predominantly located in the lungs of treated mice, indicating its potential for identifying MRSA‐induced pneumonia. The study supports the concept that bacterial surfaces can induce the self‐assembly of Rho‐FF‐Van, promoting further probe assembly due to the strong binding between the probe and bacterial surfaces. This results in stable probe accumulation at infection sites, enabling effective in vivo detection of bacterial infections.

Another study assessed the selectivity and photostability of silicon nanoparticles conjugated with Van (SiNPs–Van) for prolonged in vivo tracking of *S. aureus* infections in a mouse model.^[^ ^108^ ^]^ The probes emitted a stable signal for up to 24 h at the infection site, while the signal at the control site dissipated within 9 h. The probes could image *S. aureus* infections in vivo for up to 8 days, with the fluorescence signal correlating with the number of bacterial cells at the site of infection. This was superior to previously reported fluorescent probes, which lost signal within 21 h.^[^ ^159^ ^]^ The results indicated that SiNPs–Van probes could provide real‐time tracking of infections, offering a viable and accurate method to evaluate the effectiveness of antimicrobial treatments over an extended period.

An in vivo biodistribution study in infected mice demonstrated the selective identification of Gram‐positive bacteria using silicon nanoparticles modified with Van (DCNPs‐Van/Cu_2−*x*_Se).^[^ ^160^ ^]^ DCNPs‐Van/Cu_2−*x*_Se primarily accumulated in the liver and spleen, decreasing concentration over 7 days. The half‐life of DCNPs‐Van/Cu_2−*x*_Se in the blood suggests its potential for specific tissue imaging and continuous physiological monitoring. Histological analysis confirmed the low toxicity and good biocompatibility of DCNPs‐Van/Cu_2−*x*_Se. Dynamic near‐infrared (NIR)‐II fluorescence imaging of infected mice showed an increase in fluorescence signal at the *S. aureus* infection sites overtime, peaking at 24 h post‐injection, while the signal at *E. coli* infection sites increased slightly due to nonspecific binding (Figure 10i,j). The study concludes that DCNPs‐Van/Cu_2−*x*_Se, with its activatable fluorescence and NIR‐II luminescence, is well suited for diagnosing organ diseases, particularly in the liver and spleen, and for in vivo imaging of bacterial infections with low toxicity and good biocompatibility.

**Figure 11:**
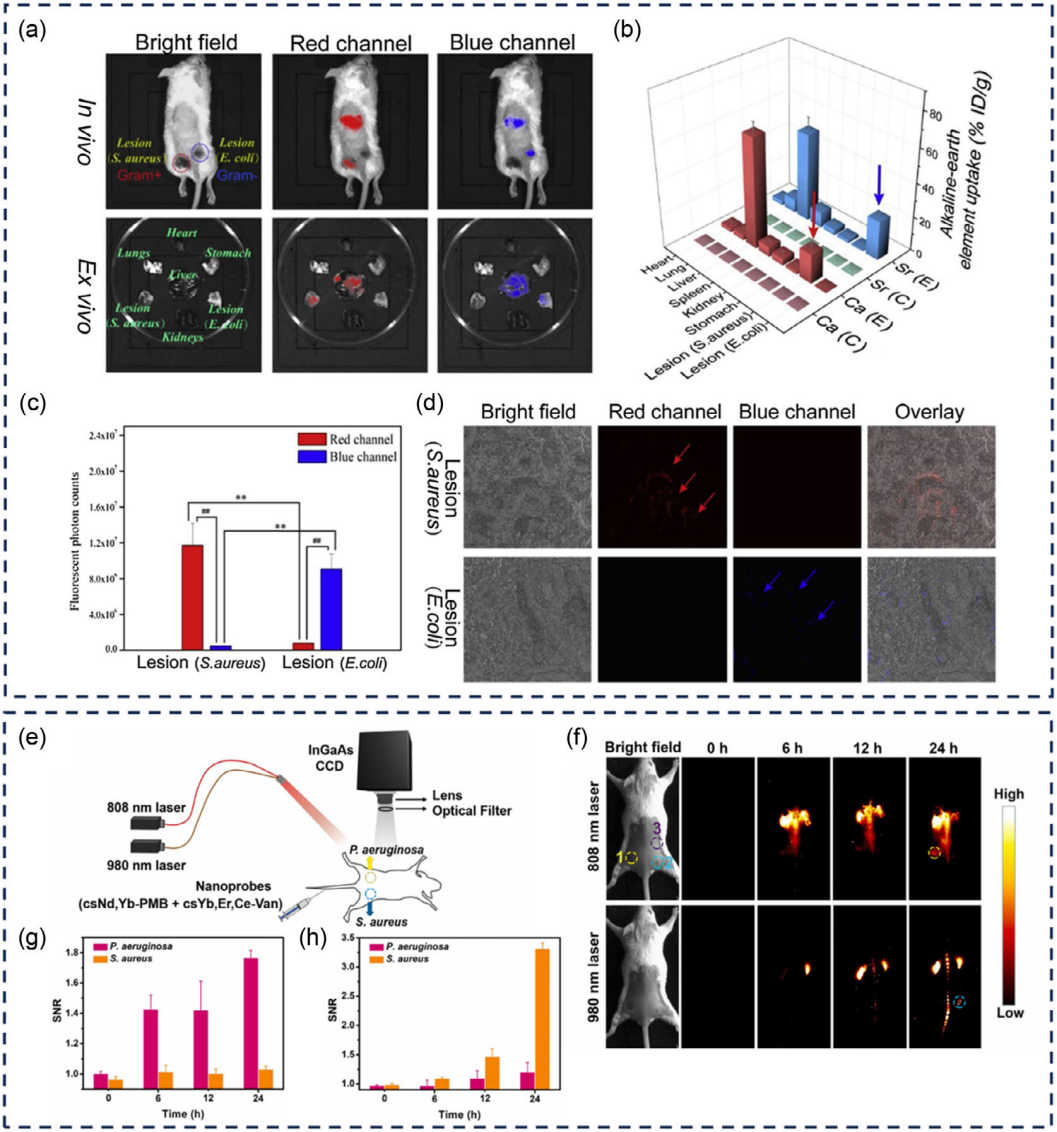
a) In vivo and ex vivo fluorescence imaging of mouse co‐infected with Gram‐positive *S. aureus* and Gram‐negative *E. coli*, with tail infection of mixed AES NPs solution. b) ICP‐MS analysis of the AES NPs biodistribution in the co‐infected mice (experimental group, E), compared to control group (C) without AES NPs injection. c) Fluorescence intensity at infection sites in the mouse co‐infected with *S. aureus* and *E. coli*. Data plotted as mean ± SD, *n* = 3, **p < 0.01, and ^##^ *p *< 0.01. d) Fluorescence images of tissue lesions from *S. aureus* and *E. coli* co‐infected mice. Images were acquired under 980 nm light excitation. Reproduced with permission.^[^ ^112^ ^]^ Copyright 2020, Elsevier. e) Schematic diagram of a multiplexed imaging device for detecting bacterial infection in mice. f) NIR‐II images of bacteria‐infected mice at different time points upon 808 or 980 nm excitation, 1) *P. aeruginosa* infected site, 2) *S. aureus* infected site, and 3) control site. Corresponding SNR in (b) upon g) 808 nm and h) 980 nm excitation. Reproduced with permission.^[^ ^113^ ^]^ Copyright 2023, Elsevier.

Tri‐doped alkaline earth sulfide NPs (AES NPs) were synthesized and covalently linked with bacterial targeting moieties, teicoplanin (TEC), and PMB, for selectively labeling and differentiating Gram‐positive and Gram‐negative bacteria.^[^^112^^]^ The functionalized AES NPs demonstrated the ability to distinguish bacterial infections from aseptic inflammation in vivo. The CaS–TEC NPs, targeting Gram‐positive bacteria, emitted red fluorescence at the infection sites, while the SrS–PMB NPs, targeting Gram‐negative bacteria, showed blue fluorescence. No fluorescence was observed at sites of sterile inflammation, indicating the specificity of these nanoparticles for bacterial infections. Additionally, co‐infected mice, injected with both *S. aureus* and *E. coli*, exhibited distinct red and blue fluorescence at respective infection sites, confirming the selective imaging capability of the nanoparticles (**Figure** **11**a,c). Distinguishable fluorescence signals observed from tissue slides of the bacterial lesion and alkaline (Ca or Sr) elemental distribution measured by Inductively coupled plasma mass spectrometry (ICP‐MS) consistently provided more evidence for the bacterial targeted effect and imaging of the two AES NPs (Figure 11b,d). Furthermore, the nonoverlapping emission bands of the two types of AES NPs enabled selective fluorescence imaging of the different bacterial strains, with intense fluorescence under 980 nm light irradiation, allowing for high sensitivity and deep tissue imaging. Subsequently, these researchers synthesized two core–shell DCNPs, NaYF4:Nd, Yb@NaYF4 (csNd and Yb), and NaYF4:Yb, Er, Ce@NaYF4 (csYb, Er, and Ce), which were surface‐modified with Van and PMB.^[^ ^113^ ^]^ The csNd and Yb–PMB nanoparticles selectively targeted Gram‐negative bacteria, showing strong NIR‐II luminescence under 808 nm excitation. In contrast, csYb, Er, and Ce–Van nanoparticles targeted Gram‐positive bacteria, exhibiting luminescence under 980 nm excitation (Figure 11e). Scanning electron microscope (SEM) imaging confirmed the selective binding of these nanoparticles to their respective bacterial targets. Mouse model studies revealed that CaS–TEC and SrS‐PMB nanoparticles could distinguish between bacterial infections and sterile inflammation (Figure 11f). For instance, the Gram‐negative *P. aeruginosa* bacteria emitted bright luminescence under 808 nm excitation, while the Gram‐positive *S. aureus* bacteria barely luminesced (Figure 11g,h). This indicates that csNd and Yb–PMB are excited explicitly by an 808 nm laser to visualize *P. aeruginosa*. At the same time, csYb, Er, Ce–Van are uniquely responsive to 980 nm excitation for detecting Gram‐positive bacteria. The SNR for *P. aeruginosa* was 1.8 at 24 h post‐injection, in contrast to 1.0 for *S. aureus*. Notably, the peak SNR for *S. aureus* reached 3.3 at 24 h with 980 nm excitation, greatly surpassing that of *P. aeruginosa*.

### 3.7 Detection of Bacteria in “Real‐World” Samples and Complex Biological Matrices

The major challenge in many bacterial detection methods is the interference caused by other biomolecules, nontarget microorganisms, and mammalian cells found in “real samples,” such as food, drinking water, wastewater, and clinical specimens like blood or urine. These samples typically undergo multiple preparatory steps to isolate bacteria, which can be time‐consuming and labor‐intensive, and may only effectively eliminate some sources of interference. This drawback requires more selective detection methods to identify bacteria within complex biological matrices. The following section will explore examples of fluorescent antibiotics successfully employed to detect and quantify bacteria in complex samples.

**Figure 12:**
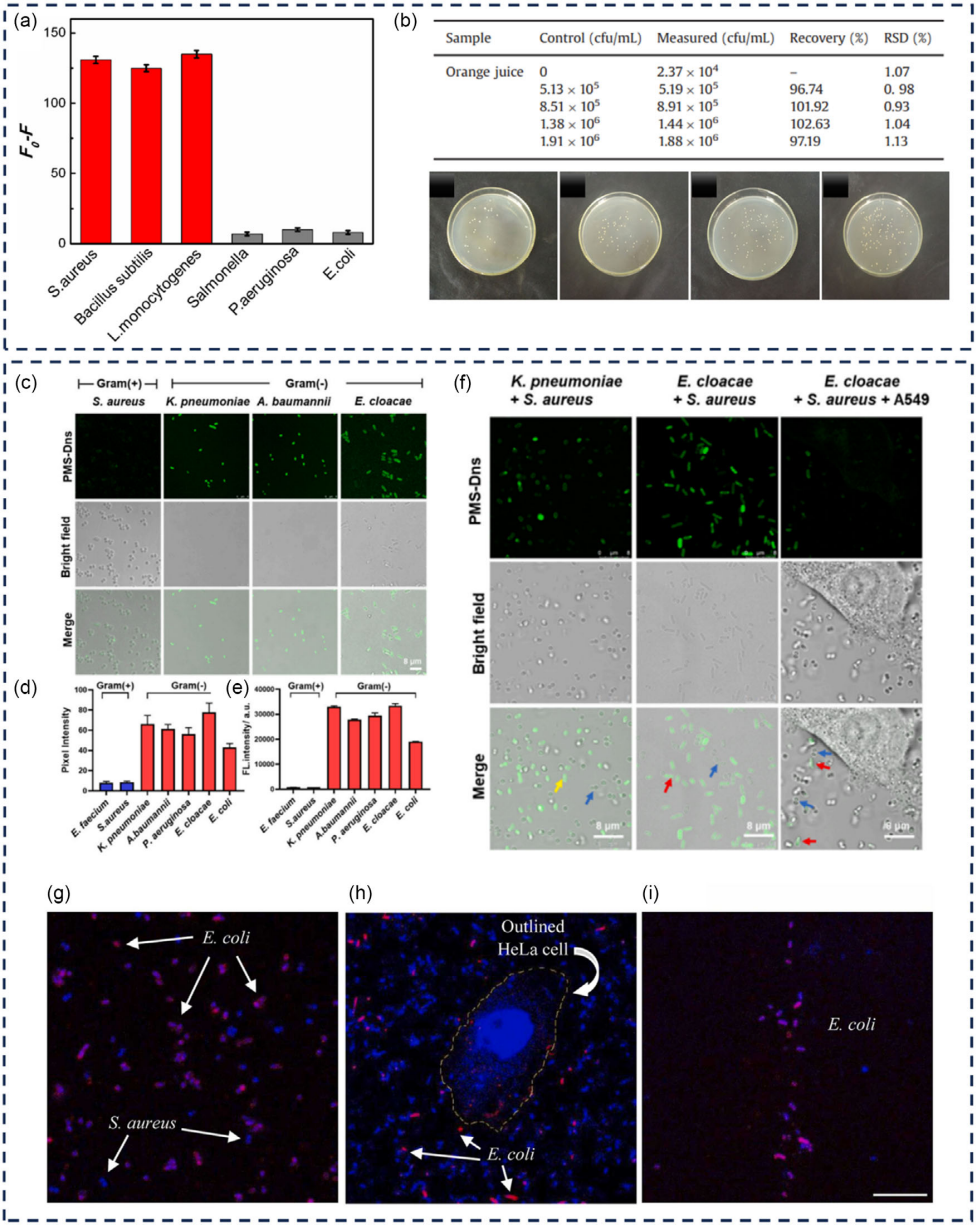
a) Decrease in fluorescence intensity (*F*_*0*_ *–F*) of CD@Van in the presence of *S. aureus*, *Bacillus subtilis*, *L. monocytogenes*, *Salmonella*, *P. aeruginosa*, and *E. coli* (each at 5.23 × 10^5^ CFU mL^−1^) Data plotted as mean ± SD and *n* = 3. b) Recoveries and traditional plate counting for different concentrations of *S. aureus* in orange juice. Reproduced with permission.^[^ ^109^ ^]^ Copyright 2015, Elsevier. c) Confocal images of *S. aureus*, *K. pneumoniae*, *A. baumannii*, and *E. cloacae* treated with PMS–DNS, with fluorescent signals captured at *λ*_ex_ = 405 nm/*λ*_em_ = 450–700 nm. d) Quantification of fluorescence intensity of PMS–DNS incubated with ESKAPE pathogens and *E. coli* by confocal laser scanning microscope. Data plotted as mean ± SD and *n* = 3. e) Fluorescence intensity of ESKAPE pathogens and *E. coli* with PMS–DNS by a microplate reader (*λ*_ex_ = 405 nm/*λ*_em_ = 450–700 nm). Data plotted as mean ± SD and *n* = 3. Scale bars represent 8 μm. f) Confocal images of *K. pneumoniae*, *E. cloacae*, *S. aureus*, and A549 cells treated with PMS‐DNS (*λ*_ex_ = 405 nm/*λ*_em_ = 450–700 nm). The arrows point to *S. aureus* (blue), *K. pneumoniae* (yellow), and *E. cloacae* (red) in the merged images. The scale bar represents 8 μm. Reproduced with permission.^[^ ^129^ ^]^ Copyright 2023, Elsevier. Merged confocal images of g) mixed sample of *E. coli* and *S. aureus*, h) mixed samples of *E. coli* and HeLa cell, and i) *E. coli* extracted from contaminated lettuce samples (red: TlipPmB‐23; blue: Hoechst). The scale bar represents 5 μm. Reproduced with permission.^[^ ^162^ ^]^ Copyright 2022, Elsevier.

CDs modified with Van (CD@Van) were utilized to develop a novel technique for rapidly and sensitively detecting Gram‐positive bacteria in complex samples.^[^ ^109^ ^]^ This approach utilizes the specific binding affinity of Van for Gram‐positive bacteria's cell walls and CDs’ unique fluorescent properties. When CD@Van binds to Gram‐positive bacteria like *S. aureus*, *B. subtilis*, and *L. monocytogenes*, the fluorescence of the CDs is quenched due to aggregation on the bacterial surface, allowing for the detection and quantification of bacteria. In contrast, adding Gram‐negative bacteria did not result in any noticeable decrease, demonstrating the selectivity for sensing Gram‐positive bacteria (**Figure** **12**a). The practical applicability of this method was validated through recovery experiments detecting *S. aureus* in orange juice, achieving recoveries above 90%, indicating high accuracy and reliability (Figure 12b). This innovative approach offers a promising solution for detecting Gram‐positive bacteria in complex samples, significantly reducing detection time and minimizing sample matrix interference.

A study by Rui et al. investigated the effectiveness of colistin‐conjugated Cy3 (COL–Cy3) in PDAT against Gram‐negative bacteria, specifically *E. coli* and *S. aureus*.^[^^162^^]^ The ability to detect Gram‐negative bacteria in mixtures with Gram‐positive bacteria and the presence of mammalian cells highlighted the potential of the technology for diagnosing polymicrobial infections and its specificity. The results showed that mixing equal amounts of two Gram‐negative bacteria produced much higher signals than samples containing a single species. In contrast, adding a Gram‐positive control to Gram‐negative bacteria resulted in fluorescence intensities that were not significantly different (*p* value <0.09) from mixtures or individual samples containing only Gram‐negative bacteria. Similarly, fluorometric measurements revealed that adding H1975 or HeLa cells to *E. coli* did not cause significant differences compared to *E. coli* alone. The measured signal was negligible when only mammalian cells were incubated with the ligand as a negative control. Confocal microscopy further confirmed that COL–Cy3 labeling occurred exclusively on *E. coli*, indicated by strong fluorescence emission, while mammalian cells showed no significant fluorescence. Moreover, the assay demonstrated its ability to detect *E. coli* in serum‐spiked samples containing up to 80% serum by volume, albeit with reduced signal intensity caused by serum protein interference. The versatility of the assay and its potential for clinical diagnosis and other applications provide advantages over traditional methods, offering a simple, single‐step incubation procedure for quantitative and qualitative analyses.

Another study focused on the selectivity and efficacy of PMS‐DNS in distinguishing Gram‐negative bacteria in the presence of various bacterial strains, including ESKAPE pathogens, and in a culture of mammalian cells.^[^ ^129^ ^]^ When tested with seven bacterial strains in Tris buffer at 37 °C for 15 min, Gram‐negative bacteria exhibited significant fluorescent signals under confocal microscopy, indicating strong binding affinity by PMS‐DNS. In contrast, Gram‐positive bacteria showed only weak signals (Figure 12c–e). However, PMS‐DNS could not label polymyxin‐resistant strains, such as *K. pneumoniae (mcr‐1)* and *E. coli (mcr‐1)*, suggesting limitations in its scope of detection. Examination of the bacterial cell envelope after PMS‐DNS treatment showed mostly intact structures, with only partial damage observed in some bacterial membranes after 30 min. The selectivity was further assessed in a co‐culture system that included Gram‐positive *S. aureus*, Gram‐negative *K. pneumoniae* or *E. cloacae*, and mammalian lung adenocarcinoma A549 cells (Figure 12f). CLSM revealed that upon treatment with 10 μm PMS‐DNS for 15 min, intense fluorescence signals were observed in the Gram‐negative bacteria *K. pneumoniae* and *E. cloacae* but not in the Gram‐positive *S. aureus* or mammalian A549 cells. These results underscore the potential of PMS‐DNS as a selective and safe tool for the specific detection of Gram‐negative bacteria in complex biological samples, including those containing a mixture of bacterial species and mammalian cells.

A thermoswitchable liposomal conjugated PMB (TLipPmB‐23) was used for detecting Gram‐negative bacteria in complex samples, including mixtures of Gram‐negative and Gram‐positive bacteria, biological samples containing mammalian cells, and food samples.^[^ ^162^ ^]^ Initial tests showed TLipPmB‐23 binds to *E. coli*, enhancing fluorescence at temperatures above 45 °C, but not to the Gram‐positive *S. aureus*. Further tests confirmed the method of selective tagging of Gram‐negative bacteria with increased fluorescence at 55 °C. The thermoswitchable nature allowed for significant detection, even with biofilms present, which usually hinders binding. The detection limits for *E. coli* were 10^5^ and 10^7^ CFU mL^−1^ for *P. aeruginosa* and *P. mirabilis*. In a mixed bacterial culture containing *E. coli* and *S. aureus*, confocal microscopy imaging confirmed that TLipPmB‐23 is selectively bound to *E. coli*, evidenced by the fluorescent labeling of only the rodlike *E. coli* bacteria (Figure 12g). Further testing with HeLa cells spiked with *E. coli* demonstrated that TLipPmB‐23 preferentially tagged *E. coli* with minimal nonspecific binding to mammalian cell membranes, attributed to the short incubation time of 1 min, which prevented uptake by mammalian cells (Figure 12h). Lastly, in food samples spiked with *E. coli*, TLipPmB‐23 successfully generated significant fluorescent signals from trace amounts of bacteria, with minimal interference from residual plant matter in the samples (Figure 12i). These results indicate that TLipPmB‐23 can effectively detect Gram‐negative bacteria like *E. coli* in various complex samples, highlighting its potential as a versatile detection platform for food safety and clinical diagnostics.

### 3.8 Detection of Bacteria in Human Organ Models

Endoscopy is a minimally invasive procedure performed through natural body openings or small surgical incisions. It aids in diagnosing and treating various conditions by allowing clinicians to visually examine organs or tissue for signs of disease, such as inflammation, ulcers, tumors, and infections. Combining endoscopy with fluorescent probes for bacterial detection enhances the precision and efficacy of diagnostic procedures in medical practice. This innovative approach allows for real‐time visualization of bacterial infections, providing direct insights into the affected areas that traditional diagnostic methods may not offer.

**Figure 13:**
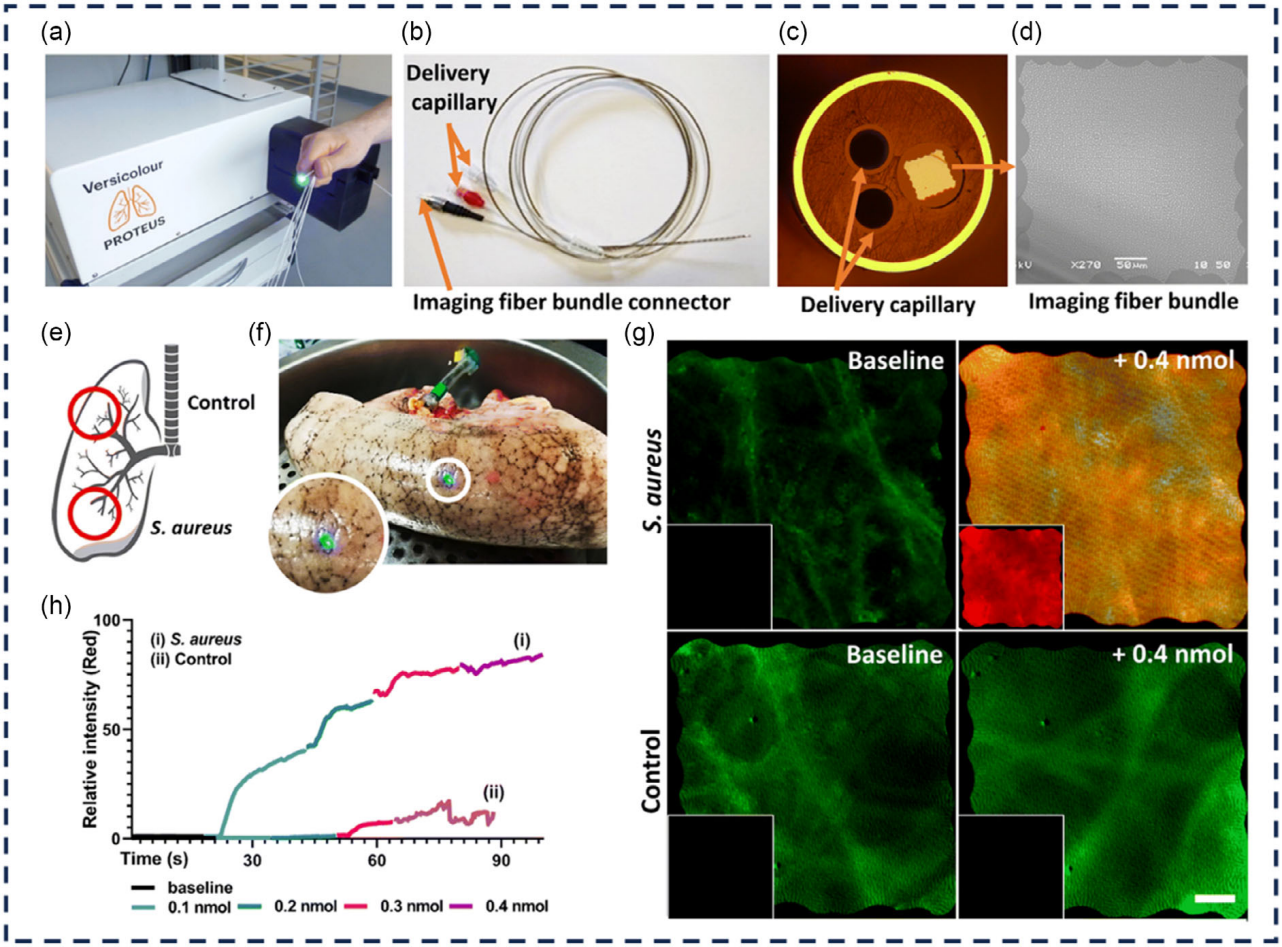
Merocy–Van selectively labels *S. aureus* in an ex vivo human lung model. a) Endomicroscopy fluorescence imaging system was used. b) The packaged three‐in‐one fiber‐based endomicroscopy device. c) End view of the distal tip of the fiber‐based endomicroscopy device. d) A micrograph of the imaging fiber with 8100 cores. e) Diagram illustrating human lungs and infection/imaging sites. f) Image of an ex vivo human lung undergoing ventilation with the imaging device inserted within the distal lung (blue light within the white circle). The inset shows a blown‐up of the device's distal end location (blue light). g) Representative images of real‐time Merocy–Van (1 μm) delivery into human ex vivo lung (baseline and following 0.4 nmol delivery shown) within *S. aureus* (top) and control (bottom) lung lobes. Green indicates lung autofluorescence. Red indicates activated Merocy–Van. Each large panel shows a two‐color overlay, with only insets showing the red channel. Scale bar = 50 μm. h) Average red fluorescence intensity per frame during real‐time delivery of 0.4 nmol Merocy–Van (1 μm) into *S. aureus* and control lung lobes. Reproduced with permission.^[^ ^135^ ^]^ Copyright 2020, Springer Nature.

In an evaluation using an explanted ventilated human lung model, Merocy–Van and an optical fiber‐based endoscope were tested for their ability to detect bacterial infections indicative of ventilator‐associated pneumonia (VAP).^[^ ^135^ ^]^ *S. aureus* was introduced into the right lower lobe, a common site for VAP, while *P. aeruginosa* served as a negative control in separate lobes (**Figure** **13**e,f). The bacterial inoculum matched clinical pneumonia diagnosis thresholds, with bronchoalveolar lavage (BAL) retrieval of ≈10^4^ CFU mL^−1^. Optical endomicroscopy imaging utilized a wide‐field system with LED and filter sets for tissue autofluorescence and Merocy–Van activation detection (Figure 13a–d). During the procedure, 500 μL of Merocy–Van (1 μm) was administered through the endoscope into the imaging area, allowing real‐time visualization of probe activation. In all experiments, a significant increase in red fluorescence was observed in the *S. aureus*–infected lung segments upon Merocy–Van application, with an average 20‐fold increase within 60 s compared to control lobes, while the green channel intensity remained constant (Figure 13g,h). Post‐imaging BAL confirmed *S. aureus* presence only in the infected lobes, with 10^4^ to 10^5^ CFU mL^−1^ recovered, demonstrating the effectiveness in selectively detecting *S. aureus* infections in lung tissue.

The potential of NBD–PMX, a fluorescent probe (SmartProbe), for diagnosing bacterial infections in chronic lung conditions was evaluated in two patient groups: those with bronchiectasis and mechanically ventilated ICU patients suspected of having pneumonia.^[^ ^163^ ^]^ In the bronchiectasis group, six patients underwent bronchoscopy, where NBD–PMX was administered, leading to immediate imaging of the distal airways and alveoli. The study reported no serious adverse events, with minor incidents like coughing attributed to the procedure. Post‐imaging BAL and sputum cultures facilitated the identification of bacterial species present. The imaging successfully detected Gram‐negative bacteria, with three patients (D2, D5, and D6) showing positive bacterial signals for pathogens like *P. aeruginosa* and *H. influenzae*. In contrast, three others (D1, D3, and D4) showed negative signals, growing either Gram‐positive or polymyxin‐resistant Gram‐negative organisms. Notably, bacterial signals were observed in regions typically considered low in bacterial burden, indicating the probe's effectiveness in real‐time detection of infections. Similarly, in an ICU patient group, the feasibility, safety, and detection capability of NBD–PMX in six patients with pulmonary infiltrates and suspected pneumonia were assessed. No serious adverse events were noted, such as those in the bronchiectasis group, and the procedure time averaged 14.7 min. Two patients (D7 and D11) displayed bacterial signals and cellular infiltrates, with patient D11 showing low levels of *Klebsiella oxytoca* and *Candida albicans* in the BAL. Other patients showed no bacterial labeling or inflammatory infiltrate, with findings suggesting atelectasis or airway colonization rather than pneumonia. Additionally, one patient (D12) was identified with a pulmonary abscess, demonstrating the probe's utility in detecting bacterial presence and alveolar cellular infiltration. These preliminary studies across two patient cohorts underscore the clinical potential of NBD–PMX as a point‐of‐care technology for the early detection of lung infections. The ability to qualitatively identify bacterial presence and alveolar cellular infiltrate in real‐time offers a promising tool for diagnosing infections in chronic suppurative pulmonary diseases and critically ill patients, enhancing the precision and efficacy of diagnostics and treatment for bacterial infections.

Van–NBD (Van–green) was developed to detect Gram‐positive infections rapidly and specifically from ocular samples.^[^ ^137^ ^]^ The study validated Van–NBD using 15 smear‐positive patient specimens and compared them with traditional Gram stain results. The findings revealed that all specimens identified as Gram‐positive bacteria by Gram staining and culture were also positively stained by the NBD probe. In contrast, Gram‐negative bacteria exhibited little to no staining with the probe. There was a 100% match in detecting Gram‐positive infections, namely, *Staphylococcus, Streptococcus, Actinomycetes spp*, and *Corynebacterium spp*. Specimens that cultured *P. aeruginosa, Neisseria subflava*, and *Klebsiella oxytoca* did not take up the NBD stain, indicating its selectivity for Gram‐positive bacteria. Such probes are ideally suited for use as a preliminary diagnostic utility in daily clinical practice (e.g., wound infection, oral infection), which normally requires accurate information on the Gram status of the bacterial etiology to tailor appropriate antimicrobial therapy. As Gram staining generates a high rate of false negative results where the sample volume is minimal, these rapid and accurate wash‐free Gram‐selective probes could potentially become the alternative future gold standard to detect and identify bacteria in clinical specimens.

## 4 Conclusion and Outlook

Fluorescent antibiotics are increasingly recognized as valuable research tools across various domains, but their full potential remains largely untapped. The versatility of these fluorescent probes, which can be tailored according to their mode of action and fluorescent properties, makes them a potential utility in many sectors ranging from microbiological research and biomedical diagnostics to industrial sectors such as food and beverage safety. These fluorescent probes have enabled researchers to elucidate antibiotic mechanisms of action, paving the way for generating novel antibiotics and antibacterial agents. They have also been used to track antibiotic uptake in cells and organisms and have shown potential as rapid, selective, and effective means of bacterial detection in vitro, in vivo, ex vivo, and in complex samples. When combined with photosensitizers or fluorophores of similar properties, selective photoimaging and photodynamic treatment of specific bacterial infections can potentially eradicate resistant bacteria and their biofilms effectively.

However, the number of available fluorescent antibiotics remains limited, and many existing antibiotics have recently become outdated. Thus, expanding the portfolio of fluorescent analogs for major antibiotic classes while retaining their potency is a critical area for future research. Although many fluorescent antibiotics have been well synthesized and evaluated for their potential, only a few have progressed to real‐world applications. This is partly due to the lack of well‐established concepts regarding their fluorescent mechanisms (e.g., ON, ON–OFF–ON, ON–OFF, and OFF–ON systems). For example, the idea of smart fluorescent probes, which remain inactive until they encounter target bacteria and then become highly active, is innovative but largely theoretical at this stage. Exploring this concept further could enable bacterial infections to be identified early via real‐time visualization.

Several challenges must be addressed before fluorescent antibiotics can be fully integrated into commercial products or applied in clinical settings. These include sustainability, consistency, reliability, and biocompatibility. Despite their ability to rapidly and selectively detect bacteria in different circumstances, only a few can meet all clinical and regulatory requirements. Addressing practical limitations, such as tissue penetration, in vivo toxicity, scalability, and dependence on specialized imaging equipment, will be critical to advancing their clinical applications. One major issue in detecting bacteria beneath the skin or in tissue is dealing with tissue scattering and absorption properties that hinder applications involving optical techniques.^[^ ^164^ ^]^ Light scattering could lead to the loss of light directionality, resulting in the loss in resolution and consequently blurring the image. Melanin and blood in tissue may reduce light intensity, leading to a low SNR.^[^ ^165^ ^]^ Several NIR fluorescent probes have been developed, allowing adequate in‐depth tissue penetration, reducing interferences from tissue autofluorescence, and improving image quality. Furthermore, strict clinical assessments remain a significant hurdle due to concerns over toxicity. These novel compounds might interact with other cells or tissues, causing side effects, trigger immune responses such as allergic reactions, or accumulate in the body due to inadequate metabolism and elimination, resulting in potential toxicity. Although extensive preclinical studies have been conducted to assess the potential toxicity of these compounds in animal models, it is still challenging to accurately know how these compounds will behave in the complex environment of the human body. Hence, developing more biocompatible and less toxic analogs and formulations of these compounds is crucial for their safe and effective use. Another major challenge is the scale‐up of fluorescent antibiotics from laboratory prototypes to commercial production, which brings new obstacles. These include manufacturing complexity, regulatory approval, cost constraints, stability and shelf life, supply chain management, and, most importantly, producing fluorescent probes with lasting efficacy against escalating drug resistance and ensuring they are a worthwhile investment and production remains streamlined and sustainable. However, many conjugation reactions occur randomly across precursor sites, resulting in heterogeneous probe populations. In contrast, controlled methods like click chemistry and SPPS allow for more precise outcomes, offering greater precision and consistency. Refining the later strategies may overcome existing limitations and pave the way toward the clinical and commercial success of fluorescent antibiotics.

Future research should also explore alternative bacterial‐targeting strategies beyond conventional antibiotics, such as antimicrobial peptides, bacteriophages, and antibodies, in combination with emerging technologies like bioinformatics and CRISPR‐Cas. Integrating fluorescent antibiotics into “pocket” devices like microfluidic chips or wearable electrochemical sensors would be intriguing, allowing rapid and on‐site bacterial detection and analysis. With continued innovation and overcoming key practical and regulatory challenges, fluorescent antibiotics hold immense promise for transforming diagnostics infection control and becoming a cornerstone of next‐generation antibacterial strategies.

## Conflict of Interest

The authors declare no conflict of interest.

## Author Contributions

**Tan Phuoc Ton**: conceptualization (lead); writing—original draft (lead); writing—review and editing (lead). **Richard Bright**: conceptualization (equal); project administration (equal); supervision (equal); validation (equal); visualization (equal); writing—original draft (equal); writing—review and editing (equal). **Vi Khanh Truong**: conceptualization (equal); project administration (equal); supervision (equal); validation (equal); visualization (equal); writing—original draft (equal); writing—review and editing (equal). **Krasimir Vasilev**: conceptualization (lead); funding acquisition (lead); project administration (lead); supervision (lead); validation (lead); visualization (lead); writing—original draft (lead); writing—review and editing (lead). **Tan Phuoc Ton** and **Richard Bright** contributed equally to this work.
